# Single‐Cell Dissection of Microglial Dysfunction in Diabetes‐Associated Neurodegenerative Vulnerability

**DOI:** 10.1002/advs.202600023

**Published:** 2026-10-28

**Authors:** Jizhou Zhang, Tsz Kwan Yau, Joyce Tse, Tsz Ho Li, Dongjie Hu, Yong Juan Zhao, Ting‐Fung Chan

**Affiliations:** ^1^ School of Life Sciences The Chinese University of Hong Kong Hong Kong China; ^2^ State Key Laboratory of Agrobiotechnology The Chinese University of Hong Kong Hong Kong China; ^3^ Division of Biomedical Health Sciences School of Medicine The Chinese University of Hong Kong, Shenzhen Shenzhen Guangdong China

**Keywords:** diabetes, microglia, neurodegeneration, single‐cell multi‐omics

## Abstract

The molecular mechanisms linking type 2 diabetes to increased neurodegenerative risk remain poorly defined. Given the critical involvement of microglial dysfunction in neurodegeneration, we constructed a temporal single‐cell atlas integrating transcriptomic and epigenomic profiles of microglia from diabetic (db/db) and control (db/m) mice across the lifespan. We delineated a nonlinear, diabetes‐specific pathological trajectory characterized by early inflammatory activation, a mid‐stage inflection in neurodegeneration‐associated signaling and metabolic stress, and late‐stage dysfunction. Chromatin accessibility profiling revealed early and persistent epigenomic priming of neurodegeneration‐associated pathways, accompanied by stage‐specific enhancer rewiring. Pseudotime analysis identified a disease‐associated microglial (DAM) state as a candidate transition node, characterized by biased polarization and accelerated maturation under diabetic conditions. Targeted validation using freshly isolated microglia and primary microglial cultures further supported the early expansion of the DAM population, a bias toward homeostatic‐to‐DAM activation, and *Arhgap15*‐dependent regulation of phagocytic activity. Together, these findings identify microglial states and candidate regulatory pathways that may link diabetes to neurodegenerative vulnerability.

## Graphical abstract

Using single‐cell transcriptomic and epigenomic profiling, researchers mapped microglial dysfunction across the lifespan of db/db mice. The authors uncovered a nonlinear microglial remodeling featuring early inflammatory priming, a mid‐stage transcriptional inflection, and late dysfunction. Under diabetic conditions, microglia exhibited biased polarization toward disease‐associated states and accelerated maturation, nominating candidate molecular regulators linking metabolic stress to neurodegenerative vulnerability.

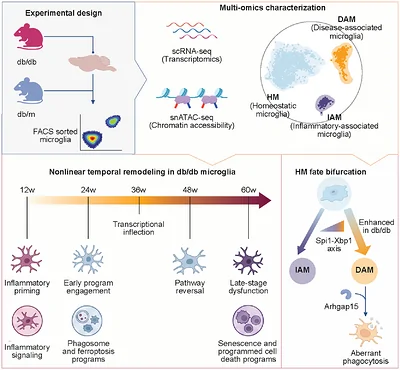

## 1 Introduction

Diabetes mellitus, particularly type 2 diabetes (T2D), is a major health challenge with a rapidly increasing prevalence worldwide [1]. Beyond its well‐established systemic metabolic effects, T2D is increasingly recognized as a significant risk factor for age‐related cognitive decline and neurodegenerative disorders, most notably Alzheimer's disease (AD) [2, 3]. Studies indicate that individuals with T2D have a 50%–100% higher risk of dementia than those without diabetes [4, 5]. Despite this strong clinical association, the molecular mechanisms that connect metabolic dysfunction to central nervous system (CNS) pathology remain poorly understood [6], limiting the development of effective neuroprotective strategies for this growing patient population.

Microglia, the resident myeloid cells of the CNS, are essential for maintaining brain homeostasis and perform critical functions such as synaptic pruning, immune surveillance, and the clearance of cellular debris [7, 8]. However, their dysregulation has been increasingly implicated in neuroinflammation and the pathogenesis of various neurodegenerative diseases [9, 10]. Growing evidence indicates that chronic hyperglycemia, insulin resistance, and systemic inflammation, hallmarks of diabetes, can considerably alter microglial activation states and phenotypic responses [11, 12, 13]. In addition, microglia exist not as a uniform population but as a heterogeneous continuum of states dynamically shaped by age, genetic factors, and the surrounding microenvironment [14, 15]. Understanding how diabetes reshapes this microglial landscape across the lifespan is therefore crucial for elucidating its neurological consequences.

The leptin receptor–deficient (db/db) mouse model provides distinct advantages for studying microglial dynamics in diabetes‐associated neurodegenerative vulnerability. Unlike diet‐induced models, db/db mice develop spontaneous, genetically programmed metabolic dysfunction that results in early‐onset obesity, severe hyperglycemia, insulin resistance, and systemic inflammation, modeling several features of human T2D progression [16]. Notably, db/db mice exhibit CNS pathologies relevant to neurodegeneration, including cognitive deficits [17], blood–brain barrier dysfunction [18], and increased Aβ deposition [19] in certain contexts, providing a robust experimental platform for investigating the mechanistic links between diabetes and neurodegeneration.

We therefore applied single‐cell technologies to comprehensively characterize microglial heterogeneity and activation dynamics in db/db and nondiabetic (db/m) mice across multiple age points (12–60 weeks). By integrating epigenomic, transcriptomic, and motif data, we identified a diabetes‐specific, nonlinear progression from homeostatic disruption to neurodegeneration‐associated microglial phenotypes, characterized by early inflammatory priming, enhancer rewiring, and accelerated commitment to disease‐associated microglial (DAM) states. We further linked the *Spi1*–*Xbp1* transcriptional axis to biased microglial fate allocation and identified *Arhgap15* as a candidate regulator of phagocytic activity. Cell–cell communication analysis also nominated PROS1–AXL as a candidate pathway associated with early DAM programs. Collectively, this study provides a high‐resolution atlas of microglial alterations in a leptin receptor‐deficient model of diabetes and a framework for future mechanistic studies of diabetes‐associated neurodegenerative vulnerability.

## 2 Results

### 2.1 Characterization of the Transcriptional States of Microglia

To resolve microglial heterogeneity during diabetes progression, we performed single‐cell RNA sequencing (scRNA‐seq) on microglia isolated from db/db and db/m mice (Figure S1A). After quality control, 27 447 cells were retained and analyzed using the Seurat pipeline, which identified 16 transcriptionally distinct populations (Figures S1B–F and S2A). These populations were evenly represented across all conditions, showing no apparent bias related to genotype, time point, or batch effects (Figure S2B).

Cluster annotation based on established markers revealed 11 microglial clusters (clusters 1–11) that expressed canonical microglial genes (*Csf1r*, *Cx3cr1*, and *Tmem119*; Figure S2C). The remaining clusters comprised non‐microglial immune cells and astrocytes, whereas cluster 12 was excluded as a potential doublet because it exhibited a distinct cell‐cycle signature and a high unique molecular identifier (UMI)‐to‐gene ratio (Figure S2D). All subsequent analyses focused on these 11 microglial clusters.

We defined these 11 clusters as distinct microglial states based on their molecular signatures and functions (Figure 1A,B and Figure S2E, Table S1). MG1 represented homeostatic microglia (HM), characterized by high expression of *Cst3*, *Sparc*, *Fcrls*, and *Tmem119*. MG2 specialized in cytoskeletal remodeling, cell polarity, and signaling pathways related to morphological plasticity and migration. MG3 functioned as a signal transduction hub, showing upregulation of transcription factors (TFs) and signaling components essential for cellular communication (Figure S2F). MG4 exhibited a stress‐responsive signature that was potentially associated with oxidative stress and hormonal signaling.

**FIGURE 1:**
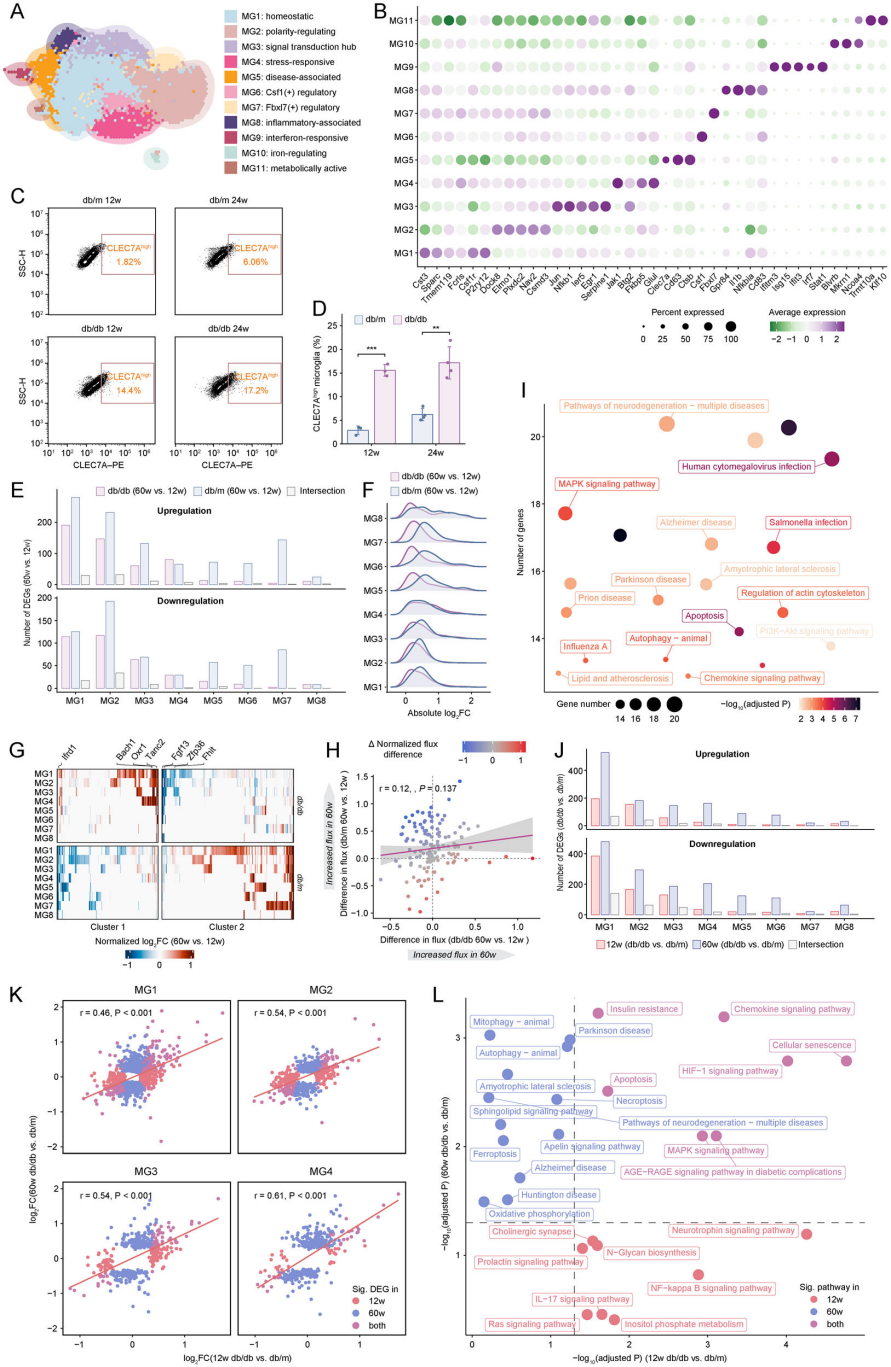
Single‐cell transcriptomic profiling reveals aging‐associated remodeling of microglia in db/db and db/m mice. (A) UMAP embeddings of microglial cells profiled through scRNA‐seq, colored by transcriptional states. (B) Dot plot showing scaled average expression (color) and percentage of expressing cells (dot size) for representative marker genes across the 11 identified microglial states. (C) Representative flow cytometry plots showing CLEC7A^high^ cells among freshly isolated CD11b^+^CD45^low^TMEM119^+^ brain microglia from db/m and db/db mice at 12 and 24 weeks. A common CLEC7A^high^ threshold, defined from the extreme upper tail of the pooled 12‐week db/m reference distribution, was applied unchanged to all samples. (D) Frequency of CLEC7A^high^ cells among parent microglia. Bars show mean ± SD, and points represent independent mice (12 weeks, *n* = 3 per genotype; 24 weeks, *n* = 4 per genotype). Two‐sided unpaired Welch's *t*‐tests; ^**^ *p* < 0.01, ^***^ *p* < 0.001. (E) Bar plot quantifying the number of upregulated and downregulated aging‐DEGs (60 weeks vs. 12 weeks) within each microglial state for db/db and db/m mice. Only clusters with > 10 cells across all samples were included. (F) Ridge plot comparing the distribution of absolute log_2_FC for aging‐DEGs in each microglial state between db/db and db/m mice. (G) Heatmap showing changes in the expression of selected aging‐DEGs across microglial states in db/db and db/m mice during aging. Red indicates upregulation and blue indicates downregulation at 60 weeks relative to 12 weeks. Representative genes are annotated. (H) Scatter plot illustrating differential flux changes of 146 metabolic pathways in db/db and db/m microglia with aging. Each point represents a pathway, colored by the delta flux change (db/db minus db/m). The Pearson correlation coefficient (r) and two‐sided *p*‐value are shown. (I) Top 20 KEGG pathways enriched among aging‐DEGs in Cluster 1 (as defined in panel G), with 14 pathways (labeled) implicated in neurodegenerative processes. (J) Bar plot showing the number of upregulated and downregulated genotype‐DEGs (db/db vs. db/m) in each microglial state at 12 and 60 weeks. Only clusters with > 10 cells across all samples were included. (K) Scatter plot showing the concordance of expression changes in genotype‐DEGs detected in MG1–MG4 between db/db and db/m mice at 12 and 60 weeks. Each point represents a gene, and the Pearson correlation coefficient (r) indicates the degree of consistency in transcriptional alterations. (L) Representative KEGG pathways enriched among genotype‐DEGs identified at 12 and 60 weeks.

We defined MG5 as a phagocytic DAM state characterized by the dominance of lysosomal and phagocytic genes, such as *Ctsb* [20] and *Clec7a* [21]. Notably, MG5 displayed the strongest enrichment of both DAM and MGnD (microglial neurodegenerative phenotype) signatures [22, 23] (Figure S2G). To orthogonally validate the presence of MG5, we quantified CLEC7A^high^ cells among freshly isolated brain microglia from db/db and db/m mice at 12 and 24 weeks. CLEC7A^high^ microglia were significantly more abundant in db/db mice at both ages (Figure 1C,D), supporting the expansion of DAM during early diabetic progression. MG6 and MG7 were defined as regulatory states, marked by *Csf1* and *Fbxl7*, respectively. MG8 and MG9 corresponded to inflammation‐associated microglia (IAM) and interferon‐responsive microglia, respectively, as defined by established markers. MG10 represented an iron‐regulating state characterized by *Blvrb* and *Ncoa4*. Finally, MG11 was enriched for pathways related to energy production and protein synthesis. Cross‐study comparison showed that our annotations captured and extended previous findings, with several states (MG3, MG5, MG8, MG9, and MG11) showing correspondence to previously reported mouse and human microglia, including disease‐associated subtypes identified in AD [24, 25, 26], highlighting the biological relevance and translational potential of our study (Figure S2H–J).

### 2.2 Transcriptional Remodeling in db/db Mice Suggests Neurodegenerative Predisposition

To delineate genotype‐specific aging trajectories, we identified aging‐associated differentially expressed genes (aging‐DEGs; 60 weeks vs. 12 weeks) across microglial clusters MG1–MG8 (> 10 cells per dataset) using three independent methods, namely the Wilcoxon test, MAST [27], and limma‐trend [28]. The strong concordance among these approaches (Figure S3A–D) supported the identification of robust sets of 456 and 823 aging‐DEGs in db/db and db/m mice, respectively (Table S2). Notably, db/db mice exhibited fewer aging‐DEGs and smaller fold‐change magnitudes (Figure 1E,F), indicating reduced transcriptional plasticity with age. This genotype‐driven disparity was further supported by the minimal overlap in aging‐DEGs (Figure 1E) and their clear separation in principal component analysis (PCA; Figure S3E), collectively revealing a unique pattern of transcriptional remodeling during aging in db/db mice.

We next focused on aging‐DEGs showing amplified genotype differences over time, defined by |Δlog_2_FC| ≥ 0.36, where Δlog_2_FC = log_2_FC(db/db) − log_2_FC(db/m) (Table S3). These genes segregated into two opposing modules: Cluster 1 included genes that were predominantly upregulated with age in db/db mice but downregulated in db/m mice, whereas Cluster 2 displayed the opposite pattern (Figure 1G). This antagonistic trend persisted across all microglial states (Figure S3F), indicating a systemic, genotype‐driven reshaping of microglial aging responses. Notably, several of the implicated genes were associated with key metabolic processes, including lipid metabolism (*Ifrd1* [29] and *Fgf13* [30]), glucose homeostasis (*Zfp36* [31]), and oxidative stress (*Oxr1* [32], *Bach1* [33], and *Fhit* [34]). Furthermore, metabolic flux analysis using scFEA [35] revealed extensive pathway rewiring (Figure 1H and Figure S3G), with over 50% of pathways showing opposing trends in db/db and db/m mice during aging (Table S4). Moreover, the narrower distribution of flux changes in db/db mice (Figure S3H) indicated restricted metabolic adaptability, consistent with the known loss of metabolic flexibility in the db/db mouse heart [36]. Functionally, the upregulated aging‐DEGs in db/db mice (Cluster 1) were significantly enriched for pathways involved in neurodegeneration and neuroinflammation (Figure 1I).

To evaluate whether these transcriptional changes translate into a program potentially relevant to neuronal damage, we performed AUCell scoring using a curated signature of secreted neuroinflammatory and neuronal stress‐associated mediators. This analysis revealed significantly higher scores in most examined db/db microglial states than in db/m controls (Figure S3I), indicating that diabetic microglia are biased toward expression of secreted mediators previously implicated in inflammatory and neuronal stress‐related processes. Collectively, these results suggest that in the context of aging, the diabetic genotype is associated with metabolic reprogramming and biases microglia toward transcriptional programs associated with neurodegenerative vulnerability.

### 2.3 Temporal Progression From Inflammation to Neurodegeneration‐Associated Microglial Programs in db/db Mice

To elucidate mechanisms underlying db/db‐driven vulnerability, we compared transcriptional profiles between db/db and db/m mice at 12 and 60 weeks using the same analytical framework (Figure S4A–D and Table S5). The analysis revealed minimal overlap in genotype‐dependent differentially expressed genes (genotype‐DEGs) between the two time points, suggesting a temporal evolution of db/db‐induced molecular perturbations (Figure 1J). This progression coincided with a marked shift in cellular contributors (Figure S4E,F). Nevertheless, the direction of DEG expression changes remained consistent over time (Figure 1K and Figure S4G), suggesting the early establishment and sustained maintenance of db/db‐driven transcriptional programs.

Functional annotations of differentially expressed genes (DEGs) revealed a coherent temporal trajectory of biological processes. At 12 weeks, the response was dominated by canonical inflammatory pathways, including interleukin‐17 and NF‐κB signaling (Figure 1L). By 60 weeks, the enrichment pattern had shifted toward pathways annotated to neurodegenerative diseases. This transition from a proinflammatory signature to a neurodegenerative profile supports a model in which the db/db genotype triggers a chronic inflammatory cascade at early stages that progressively drives microglia toward transcriptional states linked to neurodegenerative pathology.

To determine whether these findings indicate a db/db‐specific effect rather than general aging, we compared genotype‐DEGs with reported aging‐DEGs in db/m mice (60 weeks vs. 12 weeks). The results revealed minimal overlap between these two sets of DEGs (Jaccard index < 0.1 for all comparisons; Figure S4H), and the expression directions of the two DEG sets were weakly correlated (Figure S4I). These results indicated that the db/db genotype induces a unique transcriptional disruption that diverges from normal aging trajectories.

### 2.4 Single‐Nucleus ATAC Sequencing Reveals Diabetes‐Driven Chromatin Reprogramming in Microglia

To complement our transcriptomic analysis, we performed single‐nucleus ATAC sequencing (snATAC‐seq) on microglia isolated from db/db and db/m mice at 12 and 60 weeks. After quality control, 14 932 nuclei were retained (Figure S5) and partitioned into 12 chromatin states; two non‐microglial clusters were excluded based on gene activity scores (Figure S6A,B). Cross‐modality integration with the matched scRNA‐seq data revealed limited signature overlap and discordant cell‐type mapping (Figure S6C,D), reinforcing the challenges of multi‐omic alignment [26]. We therefore performed modality‐specific analyses to ensure that interpretation remained consistent with ATAC signal properties.

Peak calling with MACS2 identified 193 901 accessible chromatin regions (Table S6), of which more than 90% overlapped known DNase‐hypersensitive sites [37] and over 70% coincided with conserved cis‐regulatory elements (cCREs) from the murine brain [38, 39] (Figure S6E,F). Genomic annotation using HOMER revealed that the majority of these peaks were located in intronic and intergenic regions (>80%; Figure 2A), consistent with the enhancer‐rich chromatin architecture characteristic of brain cells [40].

**FIGURE 2:**
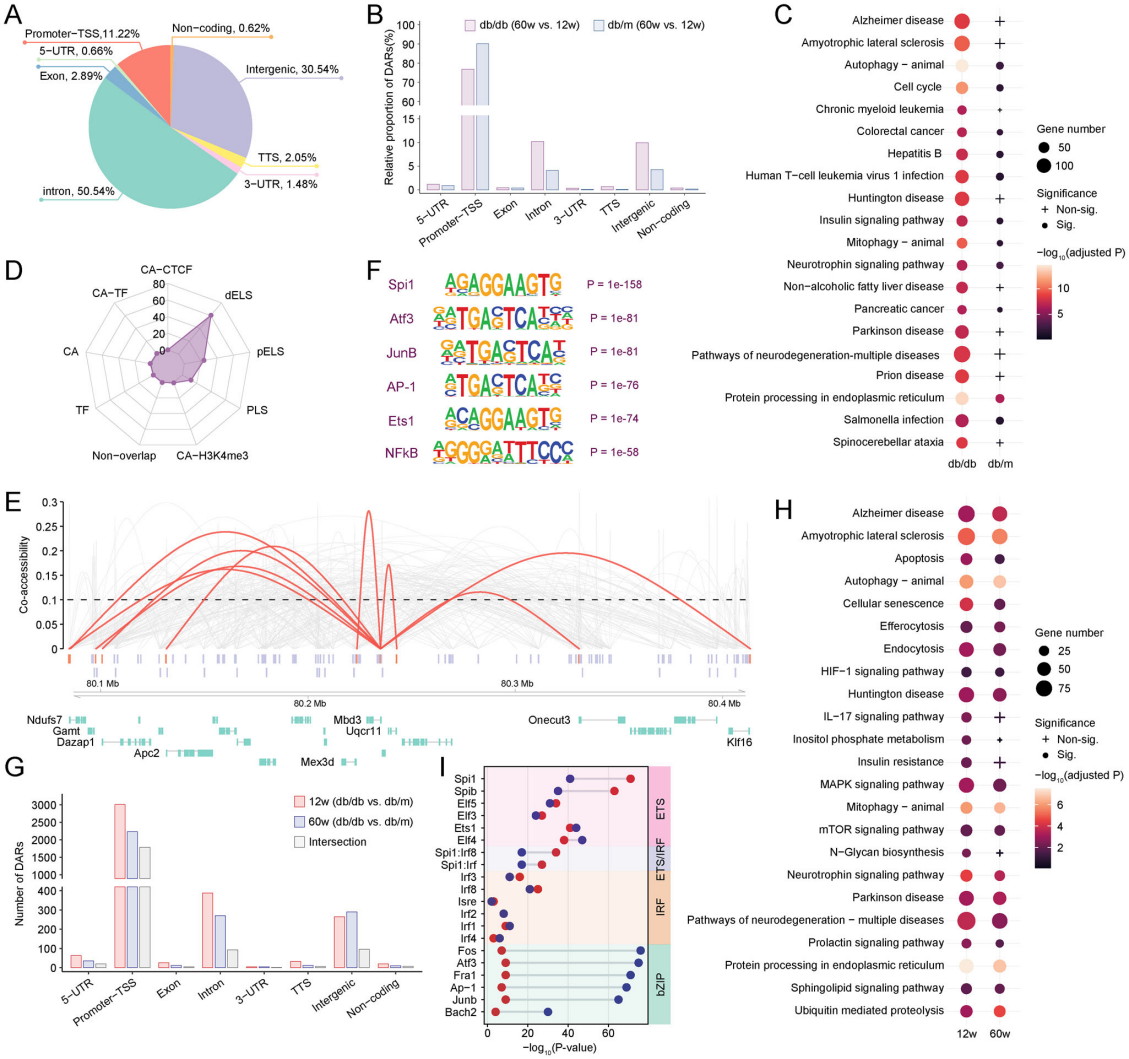
Single‐cell chromatin accessibility profiling reveals enhancer and promoter remodeling underlying microglial dysfunction in diabetes and aging. (A) Genomic annotation of accessible chromatin regions in microglial nuclei, showing the fraction of peaks overlapping annotated genomic features in the mouse genome, as determined using HOMER. TTS, transcription termination site; UTR, untranslated region. (B) Bar plot depicting the fraction of the union set of differentially accessible regions (DARs; 60 weeks vs. 12 weeks) identified in each snATAC‐seq microglial cluster for db/db and db/m mice, annotated by HOMER for genomic feature overlap. (C) Top 20 enriched KEGG pathways based on genes linked to promoter DARs. Dots and plus signs indicate significant and nonsignificant results, respectively (Benjamini–Hochberg adjusted *p* < 0.05). (D) Overlap of intronic and intergenic DARs (II‐DARs) with ENCODE cCREs. PLS, promoter‐like signature; pELS, proximal enhancer‐like signature; dELS, distal enhancer‐like signature; CA, chromatin accessible region; CA‐H3K4me3, chromatin accessible with H3K4me3 modification; CA‐CTCF, chromatin accessible with CTCF binding; CA‐TF, chromatin accessible with transcription factor (TF) binding; TF, region with TF binding but low chromatin accessibility. (E) Cis‐co‐accessibility network in ATAC‐3, illustrating an intronic DAR within the *Mbd3* locus exhibiting multiple co‐accessibility connections with promoters of neurodevelopment‐related genes (red curves); gray curves indicate background co‐accessibility. (F) Top TFs enriched at II‐DARs, as determined by motif enrichment analysis using HOMER. (G) Bar plot showing the number of union‐set DARs (db/db vs. db/m) identified in each snATAC‐seq microglial cluster at 12 and 60 weeks, annotated by HOMER for genomic feature overlap. (H) Representative KEGG pathways enriched for genes linked to promoter DARs at 12 and 60 weeks. (I) Dumbbell plot illustrating the temporal dynamics of TF family enrichment at II‐DARs in microglia at 12 and 60 weeks.

We next examined how chromatin accessibility changes with aging in db/db and db/m mice. Cluster‐specific analysis of differentially accessible regions (DARs) revealed extensive age‐related chromatin reorganization (Table S7). While proximal promoter DARs near transcription start sites (TSSs) displayed broader cellular accessibility, distal DARs were more cell type–specific (Figure S6G). Despite the overall predominance of intronic and intergenic peaks, over 75% of DARs were located in promoter‐TSS regions (Figure 2B). Functional annotation of genes associated with promoter DARs revealed a genotype‐specific pattern: pathways related to neurodegeneration, including Alzheimer's disease, prion disease, and Parkinson's disease, were exclusively enriched in db/db microglia (Figure 2C). This epigenomic profile mirrors our transcriptomic findings, indicating that diabetic microglia are epigenetically primed for neurodegenerative processes during aging.

In addition to promoter remodeling, db/db microglia exhibited a marked increase in intronic and intergenic DARs (II‐DARs), and more than 80% of these overlapped ENCODE‐annotated enhancer cCREs (Figure 2B,D). Such enhancer activation is increasingly recognized as a hallmark of microglial responses to injury, disease, and aging [41]. To explore the functional impact of these II‐DARs, we performed Cicero‐based co‐accessibility analysis [42] and mapped distal DARs to putative target promoters (Figure S6H,I). Notably, proximal–distal cCRE pairs demonstrated strong cellular specificity (Figure S6J). II‐DARs in db/db microglia showed robust co‐accessibility with promoters of neurodevelopment‐associated genes (Table S8), exemplified by an intronic DAR within *Mbd3* connected to *Apc2* [43], *Onecut3* [44], and *Uqcr11* [45] (Figure 2E). Pathway analysis of co‐accessible gene networks further confirmed enrichment for canonical neurodegeneration and stress‐response signatures (Figure S6K). Furthermore, motif enrichment in db/db II‐DARs revealed significant overrepresentation of TFs involved in microglial activation and neuroinflammation (Figure 2F and Table S9), including Spi1, Atf3, AP‐1, JunB, Ets1, and NF‐κB. These findings suggest that enhancer–promoter interactions may orchestrate the activation of neurodegeneration‐associated programs in diabetic microglia, potentially independent of promoter‐level accessibility changes.

### 2.5 Temporal Enhancer Dynamics Reveal Stage‐Specific Regulatory Rewiring

To determine how diabetes temporally affects microglial epigenomes, we performed genotype‐level comparisons at 12 and 60 weeks. Promoter‐TSS regions consistently accounted for the majority of DARs at both time points, showing substantial temporal concordance (Figure 2G and Table S10). Pathway enrichment analyses revealed stable signatures of inflammation and neurodegeneration in both 12‐ and 60‐week cohorts (Figure 2H). This stable epigenomic configuration contrasts with the phased inflammatory‐to‐degenerative transcriptional progression, suggesting that the chromatin landscape in diabetic microglia is preconfigured for pathogenic responses early in life and remains stable with aging.

In contrast, II‐DARs exhibited minimal temporal overlap (Figure 2G), suggesting stage‐specific engagement of distinct enhancer sets. To elucidate the regulatory logic underlying this enhancer turnover, we analyzed TF motifs in stage‐specific II‐DARs. We observed that II‐DARs unique to 12‐week mice were enriched for motifs belonging to the ETS and IRF families (Figure 2I), which regulate immune surveillance and inflammatory responses [46]. However, II‐DARs unique to 60‐week mice were highly enriched for bZIP family motifs, including AP‐1 and ATF subfamilies, which are central regulators of chronic stress and neurodegeneration [47, 48] (Figure 2I). These findings indicate that dynamic, stage‐specific enhancer rewiring drives microglial dysfunction throughout the diabetic lifespan.

### 2.6 Nonlinear Progression of Microglial Dysfunction in db/db Mice

To examine the progression of microglial dysfunction in db/db mice, we performed cross‐sectional profiling at 24, 36, and 48 weeks, spanning the transition from early to late aging stages. Comparisons between adjacent intervals revealed pronounced transcriptional alterations, particularly during the intermediate stages (Figure 3A and Figure S7A). Notably, the DEG burden dynamically varied across microglial clusters (Figure 3A), consistent with the known heterogeneity in microglial engagement during aging and neurodegeneration [14, 49].

**FIGURE 3:**
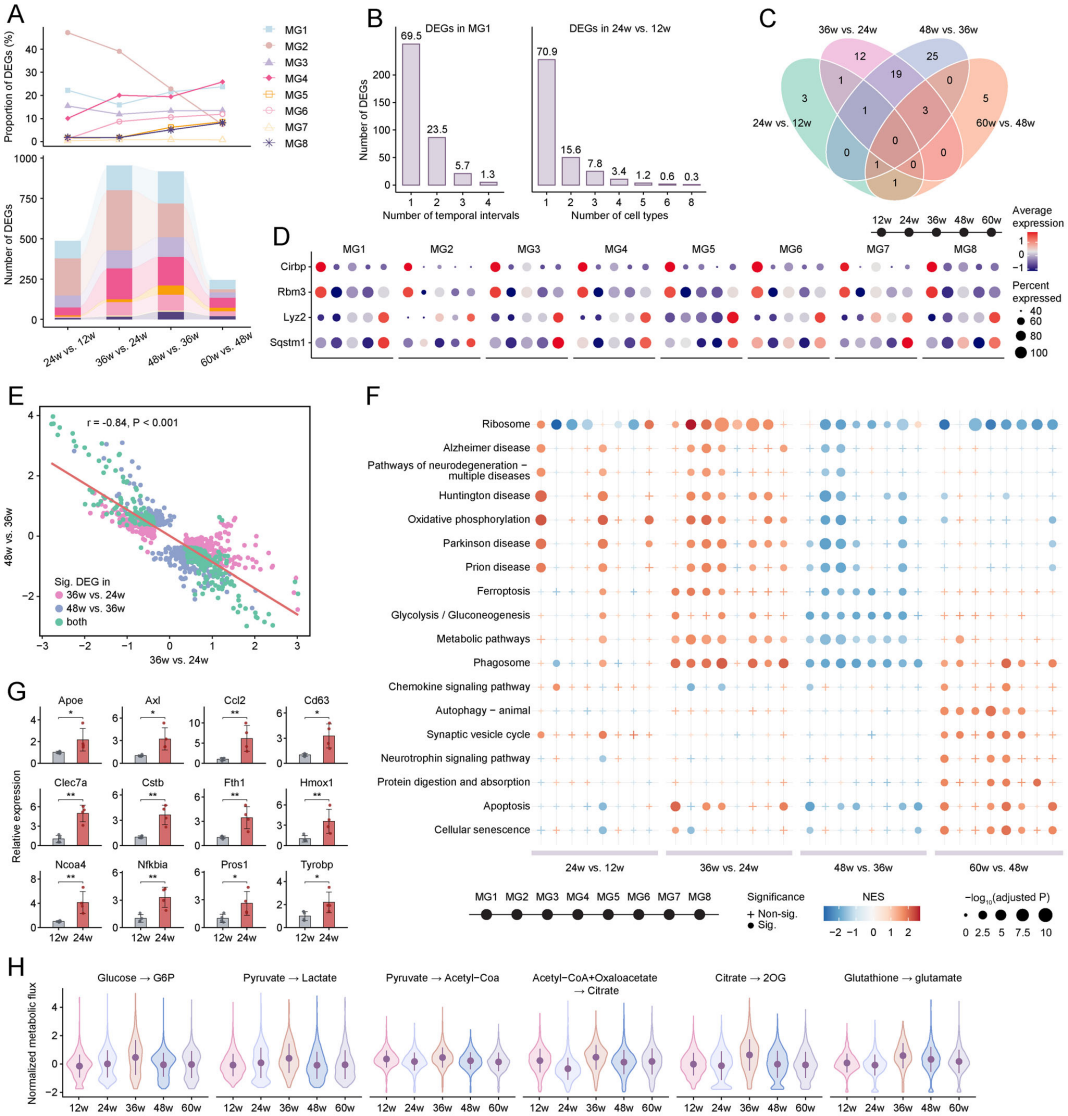
Temporal dynamics of microglial molecular programs in db/db mice. (A) Line and bar plots showing the percentage (top) and absolute number (bottom) of DEGs identified between adjacent time points across each scRNA‐seq‐defined microglial state in db/db mice. Only clusters with > 10 cells across all samples were included. (B) Left: Distribution of DEGs across all adjacent time‐point comparisons within the homeostatic microglial state (MG1). Right: Distribution of DEGs across all microglial clusters for the comparison between 12 and 24 weeks. (C) Venn diagram illustrating the overlap of shared DEGs detected across temporal comparisons. (D) Dot plot displaying scaled average expression (color) and percentage of expressing cells (dot size) for representative genes in each microglial state at different time points. (E) Scatter plot showing the concordance of DEG expression changes between the “36 weeks vs. 24 weeks” comparison and “48 weeks vs. 36 weeks” comparison. Each point represents a DEG, and the Pearson correlation coefficient (r) and two‐sided *p*‐value indicate the degree of consistency in transcriptional alterations between the two temporal windows. (F) Gene set enrichment analysis (GSEA) illustrating the dynamic engagement of representative pathways in each temporal window based on gene expression changes. (G) qRT‐PCR measurement of indicated genes in freshly isolated brain microglia from independent 12‐ and 24‐week‐old db/db cohorts (*n* = 4 per age). Expression was normalized to *Gapdh* and calculated by the 2^−ΔΔCt^ method, with the 12‐week group as the calibrator. Bars show mean ± SD, and points represent independent mice. Statistical testing was performed on ΔCt values using a two‐sided unpaired Welch's *t*‐test; ^*^ *p* < 0.05, ^**^ *p* < 0.01, ^***^ *p* < 0.001. (H) Violin plots showing normalized metabolic flux intensities for representative pathways in individual microglia across different time points.

Most DEGs exhibited high cell type and temporal specificity (Figure 3B and Figure S7B,C). We identified a subset of shared DEGs that were consistently dysregulated across multiple microglial subpopulations (≥ 5) within a single comparison, although most of them retained pronounced temporal specificity (Figure 3C). For instance, the cold‐shock protein genes *Cirbp* and *Rbm3*, both implicated in neuroprotection against stress‐induced damage [50], were downregulated at 24 weeks, indicating an early decline in stress resilience (Figure 3D). In contrast, the lysosomal enzyme *Lyz2* and the autophagy adaptor *Sqstm1* were specifically upregulated across multiple subsets at 60 weeks, suggesting a late‐stage activation of debris‐clearance pathways [51, 52] (Figure 3D). Together, these findings highlight the context‐dependent nature of microglial activation, governed by both cellular identity and developmental stage.

We observed a recurrent pattern of transcriptional reversal across the aging trajectory, evidenced by negative correlations in DEG expression changes between sequential comparisons (Figure S7D). This oscillatory regulation was particularly pronounced from 24 to 48 weeks (Figure 3E). Specifically, genes upregulated during the 24‐to‐36‐week interval frequently showed an opposing change during the 36‐to‐48‐week interval (Figure S7A–E). These bidirectionally regulated genes were predominantly enriched for inflammatory and immune processes (Figure S7F), indicating coordinated stage‐specific remodeling of microglial activation. Gene set enrichment analysis (GSEA) resolved the functional sequence underlying these temporal changes (Figure 3F). Pathways associated with neurodegenerative diseases were already activated by 24 weeks. Consistently, qRT‐PCR analysis of independent db/db cohorts confirmed significant upregulation of representative inflammatory, DAM/phagolysosomal, phagocytic‐clearance, and iron‐handling genes at 24 weeks (Figure 3G), supporting the early engagement of related programs in db/db microglia. Beyond this early phase, the 24‐to‐36‐week interval showed the strongest coordinated increase in neurodegeneration‐associated pathways, together with enhanced ribosome biogenesis, phagosome activity, and ferroptosis signaling. This response coincided with increased bioenergetic demand (Figure 3H). Many of these pathways subsequently displayed an opposing enrichment pattern in the 48‐week vs. 36‐week comparison. This rise‐and‐reversal pattern may indicate a marked inflection in microglial transcriptional programs around 36 weeks.

In the late‐stage comparison (60 weeks vs. 48 weeks), pathways associated with apoptosis, cellular senescence, protein clearance, and autophagy were positively enriched (Figure 3F). Human Phenotype Ontology [53] terms related to neurodegeneration also showed their highest activity at 60 weeks (Figure S7G), indicating progression toward a transcriptionally dysfunctional microglial state. Together, our cross‐sectional analysis revealed a nonlinear trajectory of microglial aging in db/db mice comprising early molecular activation, a mid‐stage transcriptional inflection, subsequent pathway reversal, and late‐stage microglial dysfunction.

### 2.7 Trajectory Analysis Identifies MG5 as a Candidate Pathogenic State

To reconstruct the activation dynamics of microglia in genetic diabetes, we performed trajectory inference on the integrated scRNA‐seq dataset. Unsupervised analysis using Slingshot delineated three principal lineages emerging from MG1 (Figure 4A), which were independently supported by RNA velocity (Figure S8A). Pseudotime‐resolved GSEA identified Lineage 2 as the principal pathological axis, characterized by specific positive enrichment for neurodegenerative disease ontologies, including AD, dementia, and other CNS disorders (Figure 4B). To identify the molecular events driving microglial commitment to this axis, we analyzed gene expression dynamics across lineages. This analysis revealed lineage‐specific divergence in gene clusters (Figure S8B and Table S11), with G3 and G4 showing abrupt and sustained upregulation specifically in Lineage 2 upon reaching the MG5 state (Figure 4C). Notably, these clusters were enriched for proinflammatory and immune response programs (Figure 4D). Collectively, these findings position MG5 as a candidate transition node along a neurodegeneration‐associated trajectory.

**FIGURE 4:**
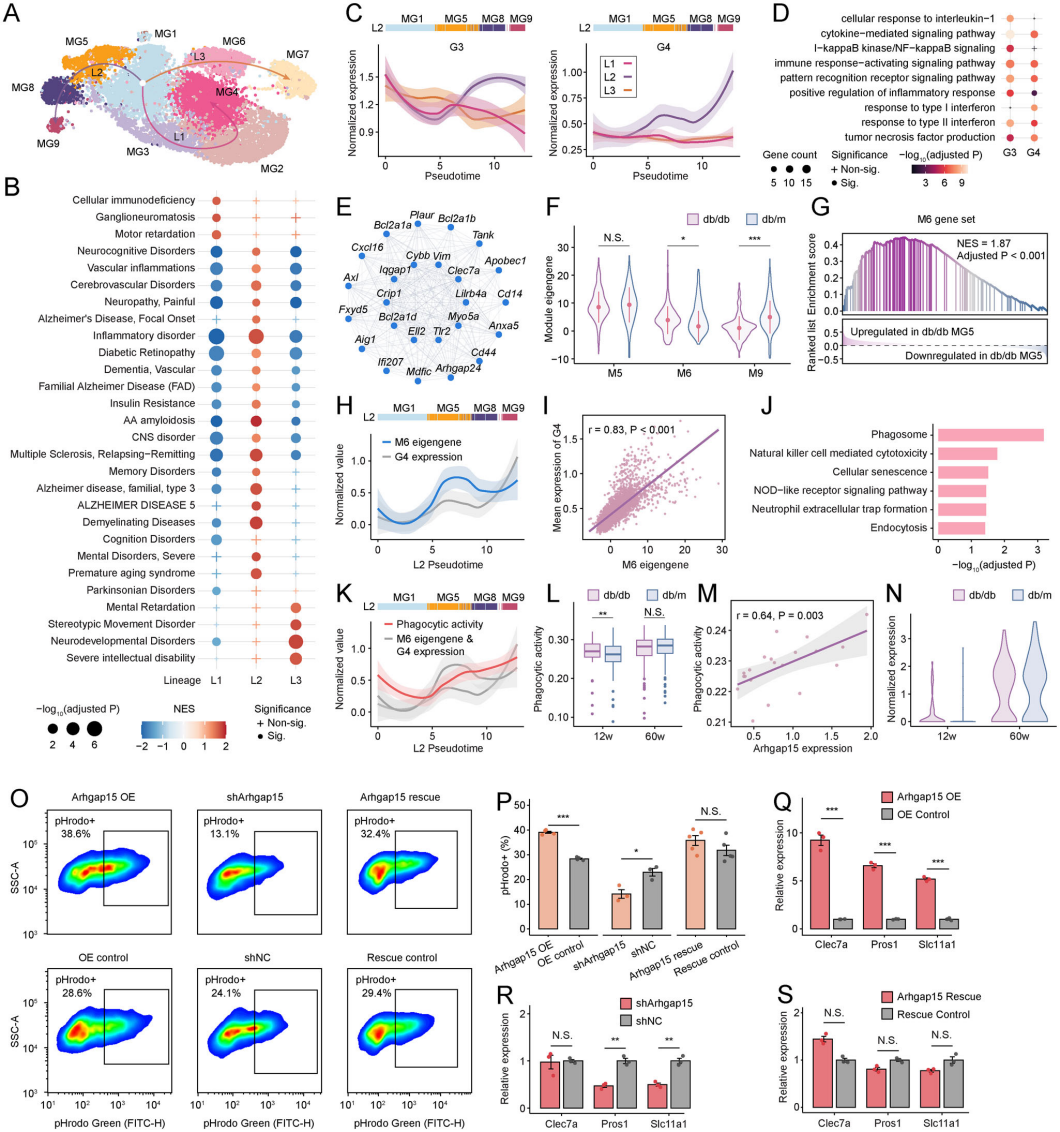
Pseudotime analysis identifies gene programs associated with phagocytic function in DAM. (A) Cell trajectory inference using Slingshot reveals three inferred developmental lineages originating from HM. UMAP visualization shows cells from the scRNA‐seq dataset colored by state, with trajectory curves overlaid. L1, Lineage 1; L2, Lineage 2; L3, Lineage 3. (B) Pseudotime‐resolved GSEA showing enrichment of CNS disorder‐related gene signatures across the three lineages. (C) Smoothed mean expression of gene clusters G3 and G4 along pseudotime for each lineage. Expression values were averaged across cells within 20 evenly sized pseudotime bins. Lines represent mean expression; shaded regions indicate 95% confidence intervals. The upper color bar denotes cells in Lineage 2, ordered by pseudotime. (D) Representative enriched Gene Ontology (GO) biological process terms for genes in clusters G3 and G4. (E) hdWGCNA module network for M6 in DAM cells, displaying the top 25 genes ranked by eigengene‐based connectivity. (F) Violin plot showing the distribution of module eigengene scores for M5, M6, and M9 in DAM cells from db/db and db/m mice. Dots indicate median values, and vertical lines within violins show interquartile ranges. ^*^ *p* < 0.05; ^***^ *p* < 0.001; N.S., not significant; Wilcoxon rank‐sum test. (G) GSEA of genes in M6 based on differential expression in DAM cells between db/db and db/m mice. (H) Smoothed trajectories showing M6 eigengene scores and mean expression of cluster G4 along pseudotime for Lineage 2. Data were binned into 20 evenly sized pseudotime intervals. Shaded regions represent 95% confidence intervals. The upper color bar denotes cells in Lineage 2, ordered by pseudotime. (I) Scatter plot showing the per‐cell correlation between G4 expression and M6 eigengene scores along Lineage 2. Each dot represents a single cell. The Pearson correlation coefficient (r) and two‐sided *p*‐value are indicated. (J) KEGG pathways enriched for overlapping genes between module M6 and cluster G4. (K) Smoothed trajectories showing phagocytic activity scores, M6 eigengene scores, and G4 expression along pseudotime for Lineage 2. Data were binned into 20 evenly sized pseudotime intervals; shaded regions represent 95% confidence intervals. The upper color bar denotes cells in Lineage 2, ordered by pseudotime. (L) Box plots comparing phagocytic activity scores in DAM cells from db/db and db/m mice at 12 and 60 weeks. ^**^ *p* < 0.01; N.S., not significant; Wilcoxon rank‐sum test. (M) Scatter plot showing correlation between phagocytic activity scores and *Arhgap15* expression across pseudotime bins along Lineage 2. Each dot represents one pseudotime bin (*n* = 20 bins). The Pearson correlation coefficient (r) and two‐sided *p*‐value are indicated. (N) Violin plots comparing *Arhgap15* expression in DAM cells from db/db and db/m mice at 12 and 60 weeks. (O) Representative flow cytometry plots of pHrodo Green *E. coli* BioParticles uptake by primary mouse microglia after *Arhgap15* overexpression (Arhgap15 OE), shRNA‐mediated knockdown (shArhgap15), or re‐expression of *Arhgap15* in knockdown cells (Arhgap15 rescue), together with the corresponding matched controls (OE control, non‐targeting shRNA [shNC], and rescue control). (P) Percentage of pHrodo^+^ cells under the conditions shown in O. (Q–S) qRT‐PCR measurement of *Clec7a*, *Pros1*, and *Slc11a1* after (Q) *Arhgap15* overexpression, (R) knockdown, and (S) rescue. Expression was normalized to *Gapdh* and calculated by the 2^−ΔΔCt^ method. In P–S, bars show mean ± SD and points represent independent cell‐culture experiments; matched conditions were compared using two‐sided paired *t*‐tests. In P, *n* = 4, 3, and 5 for overexpression, knockdown, and rescue, respectively; *n* = 3 for each comparison in Q–S. Significance: ^*^ *p* < 0.05; ^**^ *p* < 0.01; ^***^ *p* < 0.001; N.S., not significant.

We next examined whether MG5 represents a stable phenotypic state or a transient activation event. To define the core molecular identity of MG5, we performed high‐dimensional weighted gene co‐expression network analysis (hdWGCNA) [54]. This analysis identified 14 gene modules, among which M5, M6, and M9 exhibited high specificity for this state (Figure S8C,D and Table S12). M5 was anchored by ribosomal subunits, consistent with its enrichment for protein synthesis and neurodegenerative pathways (Figure S8E,F). M6 and M9 contained established MG5 markers (*Clec7a* and *Cd63*, respectively; Figure 4E and Figure S8E), underscoring their relevance to MG5 identity and functionality. Notably, M6 exhibited higher cell‐level activity in diabetic MG5 cells compared with their nondiabetic counterparts (Figure 4F,G) and showed substantial overlap and transcriptional correlation with the trajectory‐induced cluster G4 (Figure 4H,I and Figure S8G). This convergence suggests that genes activated during the transition to MG5 are maintained as a coherent module defining the MG5 state.

### 2.8 Early Phagocytic Dysregulation Links MG5 to Neurodegeneration‐Associated Programs

KEGG pathway analysis of the M6/G4‐overlapping genes revealed a strong predominance of phagocytic functions, with the phagosome identified as the top‐ranked term (Figure 4J). Although microglial phagocytosis is generally neuroprotective, its chronic or aberrant activation can lead to synaptic loss and neurodegeneration [55]. We subsequently derived a phagocytic activity score using a gene set of positive regulators defined by Gene Ontology (GO:0050766). Notably, the phagocytic activity score along Lineage 2 broadly paralleled both the M6 eigengene and G4 expression trajectories (Figure 4K), surging upon reaching the MG5 state. This aberrant phagocytic signature was already evident in db/db MG5 cells by 12 weeks (Figure 4L), suggesting that altered phagocytic programming is an early feature in diabetes‐associated microglial dysfunction.

Among the overlapping genes, we identified *Arhgap15*, which encodes a Rho GTPase–activating protein. Although ARHGAP15 negatively regulates phagocytosis in neutrophils [56], its expression here closely paralleled the phagocytic activity score along Lineage 2 (Figure 4M). Notably, *Arhgap15* expression was uniquely elevated in 12‐week‐old db/db MG5 cells compared with db/m controls (Figure 4N), temporally coinciding with early phagocytic dysregulation. K‐Nearest‐Neighbors Conditional‐Density Resampled Estimate of Mutual Information (kNN‐DREMI) analysis further showed positive expression correlations between *Arhgap15* and phagocytosis markers (Figure S8H), suggesting a context‐dependent role for *Arhgap15* in modulating phagocytosis.

To functionally examine this relationship, we next perturbed *Arhgap15* levels in primary mouse microglia (Figure S8I). Overexpression of *Arhgap15* significantly increased the uptake of pHrodo‐labeled bioparticles and elevated the expression of the phagocytosis‐associated genes *Clec7a*, *Pros1*, and *Slc11a1* (Figure 4O–Q). Conversely, shRNA‐mediated *Arhgap15* knockdown reduced bioparticle uptake and significantly decreased *Pros1* and *Slc11a1* expression, whereas the change in *Clec7a* was not significant (Figure 4O,P,R). Re‐expression of *Arhgap15* following knockdown restored phagocytic activity and the expression of all three markers to levels comparable to those in the matched control (Figure 4O,P,S). These reciprocal effects, together with the rescue experiment, support a cell‐autonomous role for *Arhgap15* in regulating microglial phagocytosis.

### 2.9 *Spi1* and *Xbp1* are Associated With HM‐to‐DAM Polarization

While the primary inferred trajectory proceeded from MG1 (HM) through MG5 (DAM) to MG8 (IAM), RNA velocity suggested an alternative, direct HM‐to‐IAM route that bypassed DAM (Figure S8A). We visualized this transition by projecting the cells onto an expression subspace defined by the DEGs of the HM, DAM, and IAM populations using CytoSimplex [57] (Figure 5A). RNA velocity further supported the divergence of HM cells toward DAM and IAM fates, hereafter termed the HM‐DAM and HM‐IAM branches, respectively, across all examined samples (Figure S9A). To experimentally examine the two transitional branches originating from HM, we performed multicolor flow cytometry to quantify marker‐defined HM‐DAM‐ and HM‐IAM‐like populations in freshly isolated brain microglia from independent db/db and db/m cohorts at 12 and 24 weeks. Both populations were detected, with selective enrichment of HM‐DAM‐like cells in db/db mice (Figure 5B and Figure S9B,C), supporting a diabetes‐associated bias toward the DAM branch.

**FIGURE 5:**
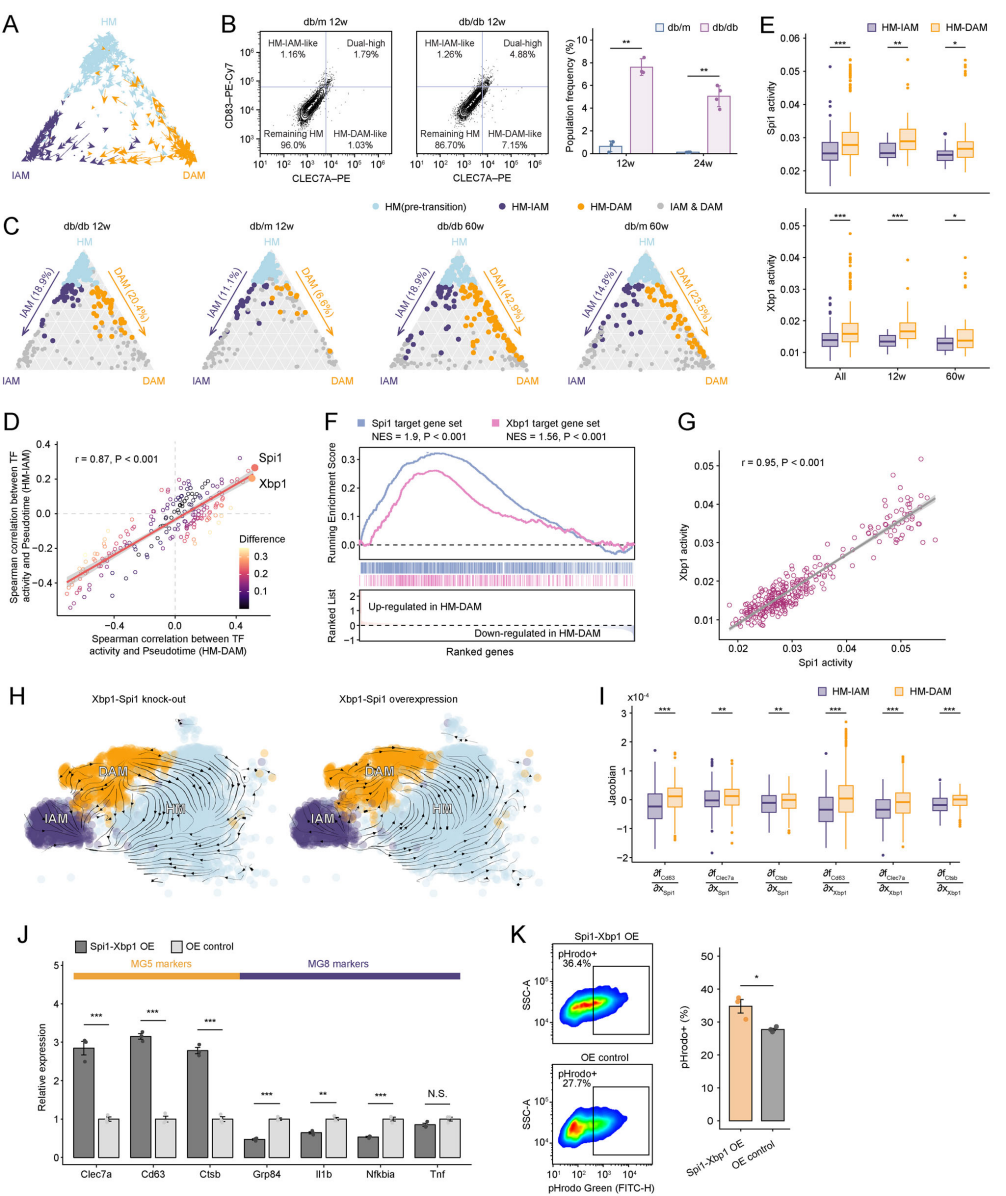
*Spi1* and *Xbp1* are associated with the transition of HM to DAM in db/db mice. (A) Ternary plot showing the distribution of HM, DAM, and IAM based on subtype‐specific gene signatures, overlaid with RNA velocity vectors illustrating transcriptional state transitions. (B) Left, representative CLEC7A‐vs.‐CD83 flow cytometry plots of P2RY12^high^ microglia from 12‐week‐old db/m and db/db mice. Fixed quadrant gates classify P2RY12^high^CLEC7A^high^CD83^low^ and P2RY12^high^CLEC7A^low^CD83^high^ cells as HM‐DAM‐like and HM‐IAM‐like, respectively. Right, frequency of HM‐DAM‐like cells among all P2RY12^high^ microglia at 12 and 24 weeks. Bars show mean ± SD, and points represent independent mice (12 weeks, *n* = 3 per genotype; 24 weeks, *n* = 4 per genotype). Statistical testing used two‐sided unpaired Welch's *t*‐tests; ^**^ *p* < 0.01. (C) Ternary plot showing the proportion of HM cells transitioning toward DAM or IAM states in db/db and db/m mice at 12 and 60 weeks. (D) Pseudotemporal correlation analysis of TF activity in HM‐DAM (*x* axis) and HM‐IAM (*y* axis) cells. Each dot represents a TF, colored by the difference in Spearman correlation coefficients between pseudotime and SCENIC score for the two trajectories. The Pearson correlation coefficient (r) and *p*‐value are indicated. (E) Box plots showing SCENIC‐inferred activity scores for Spi1 and Xbp1 in HM‐DAM and HM‐IAM cells at 12 weeks, 60 weeks, and combined time points. ^*^ *p* < 0.05, ^**^ *p* < 0.01, ^***^ *p* < 0.001; Wilcoxon rank‐sum test. (F) GSEA of *Spi1* and *Xbp1* target gene sets based on differential expression between HM‐DAM and HM‐IAM cells. (G) Scatter plot showing the correlation between Spi1 and Xbp1 activity in individual HM‐DAM cells. Each dot represents a single cell. The Pearson correlation coefficient (r) and two‐sided *p*‐value are indicated. (H) In silico gene perturbation simulations using Dynamo showing the effects of double knockout and double activation of *Spi1* and *Xbp1* on inferred microglial developmental trajectories. (I) Box plot showing the Jacobian‐based regulatory effect of *Spi1* and *Xbp1* on canonical DAM marker genes, compared between HM‐DAM and HM‐IAM cells. ^**^ *p* < 0.01, ^***^ *p* < 0.001; Wilcoxon rank‐sum test. (J) qRT‐PCR measurement of DAM and IAM marker genes in primary mouse microglia co‐expressing *Spi1* and *Xbp1* (Spi1–Xbp1 OE) or receiving the matched vector control (OE control). Expression was normalized to *Gapdh* and calculated by the 2^−ΔΔCt^ method. (K) Representative flow cytometry plots and quantification of pHrodo Green *E. coli* BioParticles uptake by primary mouse microglia co‐expressing *Spi1* and *Xbp1* or receiving the matched vector control. In J and K, bars show mean ± SD and points represent independent cell‐culture experiments (*n* = 3). Statistical testing used two‐sided paired *t*‐tests; ^*^ *p* < 0.05; ^**^ *p* < 0.01; ^***^ *p* < 0.001; N.S., not significant.

We next quantified HM fate allocation toward DAM or IAM using the scRNA‐seq data. The two branches exhibited distinct transcriptional programs (Figure S9D–F), and their relative representation varied across the sampled genotypes and ages (Figure 5C). The db/db microglia showed greater allocation to the HM‐DAM branch, particularly at 12 weeks of age, when the HM‐DAM to HM‐IAM ratio was approximately twice that in age‐matched db/m controls. In contrast, db/m microglia maintained a more balanced fate distribution. Within the inferred trajectory, the proportion of HM‐DAM cells increased steadily along pseudotime (Figure S9G), underscoring a continuous strengthening of this trajectory under diabetic stress.

To identify candidate regulators associated with HM‐DAM polarization, we employed pySCENIC [58] to infer TF activity at single‐cell resolution within the HM population. While many TFs displayed coordinated activity across both trajectories, a subset exhibited branch‐selective bias favoring the HM‐DAM lineage (Figure 5D and Figure S9H). Among these, *Spi1* and *Xbp1* emerged as candidate regulators, showing the highest pseudotemporal correlation and regulon specificity for the HM‐DAM trajectory (Figure 5D and Figure S9I). Their activity peaked at 12 weeks in db/db mice, temporally coinciding with the maximal HM‐DAM transition bias (Figure 5E). GSEA confirmed the functional relevance of *Spi1* and *Xbp1*, showing positive enrichment of their target genes within the HM‐DAM population (Figure 5F). To further evaluate their regulatory effect on DAM identity, we performed kNN‐DREMI analysis, which revealed expression dependencies between *Spi1* or *Xbp1* and DAM marker genes (Figure S9J).

In addition to their individual regulatory roles, *Spi1* and *Xbp1* exhibited coordinated activity and expression within HM‐DAM cells (Figure 5G and Figure S9K), with substantial overlap in their downstream regulons (Figure S9L). We next used Dynamo [59] to predict cell‐state shifts following genetic perturbations. *In silico* dual knockout of *Spi1* and *Xbp1* predicted a shift away from the HM‐DAM transition, whereas their co‐overexpression strongly reinforced this pathway and reduced the HM‐IAM branch (Figure 5H). Consistently, RNA Jacobian analysis revealed a stronger regulatory effect of *Spi1* and *Xbp1* on DAM marker genes during the HM‐DAM transition than during the HM‐IAM transition (Figure 5I).

To functionally examine the predicted role of the *Spi1–Xbp1* axis, we assessed its regulation of DAM‐associated features in primary mouse microglia. Co‐expression of *Spi1* and *Xbp1* significantly increased the expression of DAM signature genes and reduced the expression of several IAM‐associated markers (Figure 5J and Figure S9M). These molecular changes were accompanied by corresponding functional effects: *Spi1*–*Xbp1* co‐expression significantly increased the uptake of pHrodo‐labeled bioparticles (Figure 5K). Together with the RNA‐velocity and regulatory‐network analyses, these results support a model in which coordinated *Spi1–Xbp1* activation biases the transition of homeostatic microglia toward the DAM branch and promotes DAM‐associated transcriptional activation and phagocytic function under diabetic conditions.

### 2.10 Early Maturation of DAM in Diabetic Mice

Pseudotemporal trajectory analysis revealed a genotype‐specific divergence in MG5 progression: while MG5 cells gradually accumulated in db/m mice, their relative representation declined over pseudotime in db/db mice (Figure 6A). This observation suggests early DAM maturation followed by rapid progression into more advanced inflammatory states under diabetic conditions. Comparative transcriptomic profiling of MG5 cells from db/db and db/m mice revealed global conservation in gene expression but identified a subset of DEGs notably enriched for TFs (Figure 6B; overrepresentation test, *p* = 0.012), implicating TF‐driven remodeling of the diabetic DAM phenotype.

**FIGURE 6:**
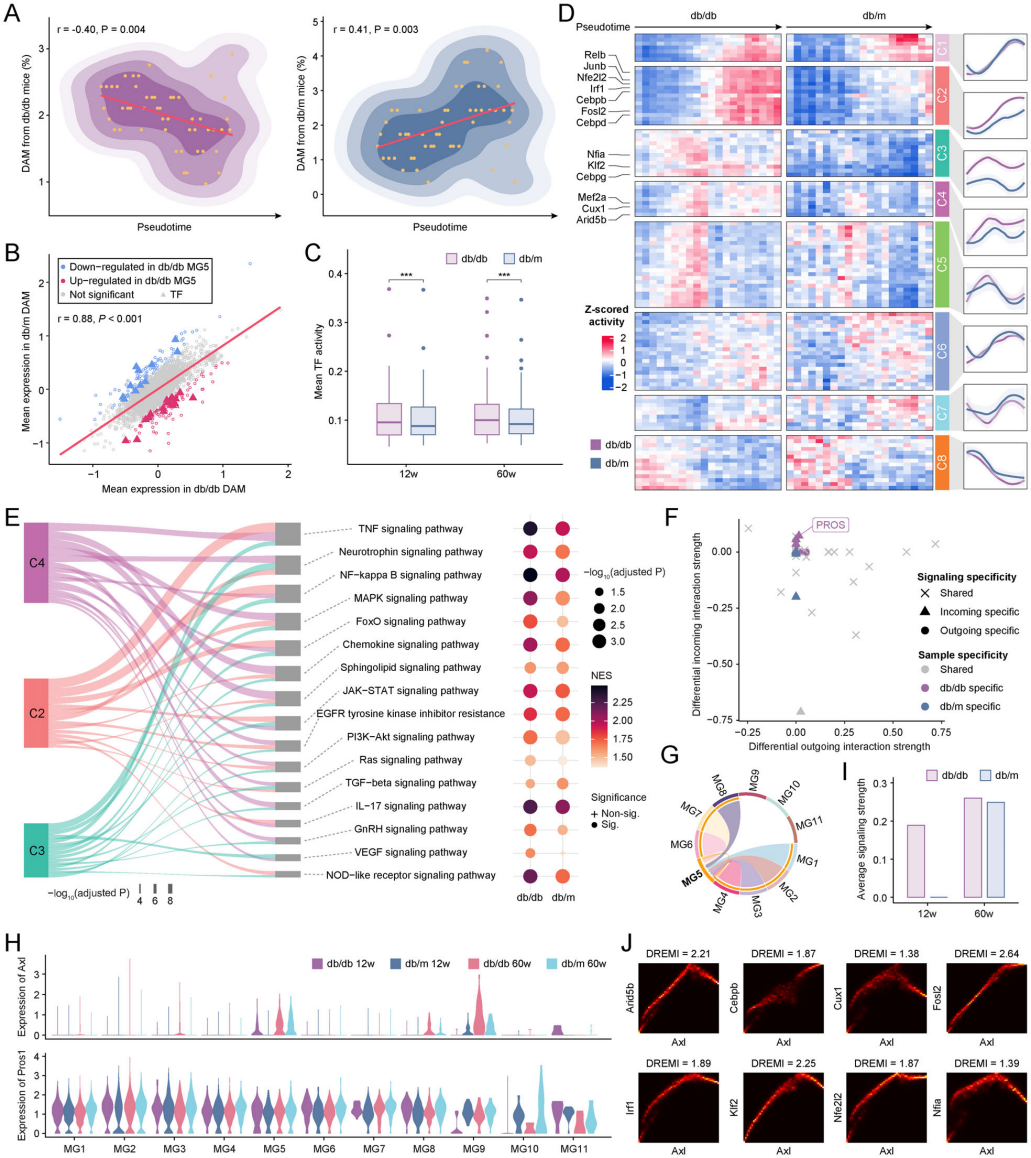
Computationally inferred signaling associated with early DAM programs in diabetic microglia. (A) Scatter plots showing the proportion of DAM cells from db/db (left) and db/m (right) mice across 50 evenly sized bins along the inferred trajectory. The Pearson correlation coefficient (r) and two‐sided *p*‐value are indicated. (B) Scatter plot comparing mean gene expression between DAM cells from db/db and db/m mice, with genes upregulated (red) and downregulated (blue) in db/db mice highlighted. Differentially expressed TFs are indicated by triangle symbols. The Pearson correlation coefficient (r) and two‐sided *p*‐value are shown. (C) Box plot showing the distribution of mean SCENIC‐inferred TF activity scores in DAM cells from db/db and db/m mice. ^*^ *p* < 0.05, ^**^ *p* < 0.01, ^***^ *p* < 0.001; Wilcoxon rank‐sum test. (D) Heatmap showing the unsupervised clustering of TF activity in DAM cells across 20 evenly sized bins along pseudotime, with TFs grouped into eight clusters. Line plot illustrates smoothed TF activity along the trajectory. (E) Left: Representative KEGG pathways enriched for target genes of TFs in clusters C2, C3, and C4. Right: Pseudotime‐resolved GSEA of these pathways in DAM cells from db/db and db/m mice. (F) Dot plot comparing the relative signaling strength of ligand–receptor pathways between DAM cells from db/db and db/m mice, with points colored by genotype specificity and shaped by signaling type. (G) Network plot showing PROS signaling pathway‐related interactions among microglial populations. (H) Violin plots showing expression of *Pros1* and *Axl* in each microglial cluster by genotype and time point. (I) Bar plot showing average inferred PROS signaling strength in DAM cells from db/db and db/m mice at 12 and 60 weeks. (J) Single‐cell kNN‐DREMI analysis (DREVI plots and DREMI values) illustrating the relationship between the expression of *Axl* and selected TFs in C2, C3, and C4 after MAGIC imputation.

The prominence of TF‐associated DEGs prompted us to systematically assess TF activity in the MG5 population. Overall TF activity was significantly increased in db/db MG5 cells compared with db/m controls at both 12 and 60 weeks (Figure 6C). Unsupervised clustering of TF activity dynamics along pseudotime identified eight clusters (Table S13), among which C2, C3, and C4 exhibited consistently increased activity in db/db MG5 cells throughout progression (Figure 6D). Functional annotation of these TF clusters, based on their target genes, revealed significant enrichment of canonical pathways central to microglial activation, differentiation, and maturation, including TNF, NF‐κB, and JAK–STAT signaling pathways [60, 61] (Figure 6E). Pseudotime‐resolved GSEA further supported the enhanced activation of these pathways in db/db MG5 cells (Figure 6E). This coordinated and sustained upregulation of TF‐driven inflammatory pathways provides a potential regulatory basis for the accelerated DAM maturation.

Emerging studies highlight the pivotal role of intercellular communication in orchestrating intracellular responses [62]. To identify candidate upstream signals associated with this transcriptional reprogramming, we next examined cell‐cell communication networks using CellChat [63]. Our analysis predicted the PROS signaling pathway as a prominent incoming signal to MG5 cells (Figure 6F). PROS1 and the receptor tyrosine kinase AXL have been implicated in triggering multiple inflammatory cascades through intracellular signaling [64, 65]. Notably, MG5 was predicted to receive PROS signaling from all other microglial subpopulations (Figure 6G).

Expression analysis revealed distinct cell‐state‐specific regulation of this axis. While *Pros1* was widely expressed, *Axl* expression was concentrated in MG5 and MG9 subpopulations (Figure 6H). At 12 weeks, PROS signaling was inferred in db/db microglia but not in age‐matched db/m controls (Figure 6I). This pattern coincided with *Axl* expression in early diabetic microglia. kNN‐DREMI analysis further revealed expression dependencies between *Axl* and the key TFs from clusters C2, C3, and C4 within the MG5 population (Figure 6J). Collectively, these computational analyses nominate a putative PROS1*–*AXL signaling pathway associated with TF activity and early DAM‐associated programs in the present dataset.

To investigate this relationship and its behavioral relevance, we used an AAV‐mediated approach intended for microglia‐preferential *Axl* knockdown *in vivo* [66, 67] (Figure S10A). No significant behavioral differences were detected between treatment groups within either genotype (Figure S10B). However, histological evaluation showed reporter expression predominantly in non‐microglial cells (Figure S10C), indicating ineffective microglial targeting, and microglial *Axl* knockdown was not verified. This technical pilot therefore did not meet the requirements for functional validation, so the results cannot be interpreted as evidence for or against a causal role for *Axl* in vivo. The PROS1–AXL pathway remains a computational candidate for future mechanistic studies.

## 3 Discussion

In this study, leveraging single‐cell multi‐omics and cross‐sectional profiling in the db/db genetic diabetes model, we delineated a high‐resolution molecular and temporal atlas of microglial dysfunction. Our integrative analysis reveals that diabetes reshapes microglial heterogeneity and function, suggesting a progressive pathological trajectory from early inflammatory and epigenetic priming to late‐stage pathogenic states.

The db/db mice did not simply recapitulate the age‐related changes observed in db/m controls but showed distinct transcriptional and epigenomic programs linked to neurodegeneration‐associated microglial remodeling. This divergence was accompanied by restrained transcriptional responsiveness, marked by a reduced number of aging‐associated DEGs and smaller expression shifts, suggesting constrained adaptive plasticity under chronic metabolic stress [68]. Notably, snATAC‐seq analysis indicated that this neurodegenerative vulnerability is epigenetically encoded before transcriptional activation, establishing chromatin remodeling as a “molecular memory” that scaffolds later pathological outputs with age and accumulating metabolic stress.

Cross‐sectional profiling revealed a nonlinear trajectory of microglial activation in diabetic mice. We demonstrated early activation of neurodegeneration‐associated programs by 24 weeks in db/db mice. The subsequent 24‐to‐36‐week interval showed the strongest coordinated increase, followed by opposing pathway changes between 36 and 48 weeks and late senescence, apoptosis, and protein‐clearance signatures. This rise‐and‐reversal pattern suggests an apparent transcriptional inflection around 36 weeks during aging in db/db mice. However, because independent 36‐ and 48‐week cohorts were not examined, this mid‐stage feature remains computationally inferred.

Trajectory analysis positioned the phagocytic DAM state (MG5) as a candidate transition state along a neurodegeneration‐associated trajectory. Within this state, our findings revealed a context‐dependent role for *Arhgap15* in phagocytosis. Although ARHGAP15 has been characterized as a RacGAP that suppresses phagocytosis in neutrophils [56], our data demonstrate that in primary mouse microglial cultures, *Arhgap15* can facilitate phagocytosis. We hypothesize that the metabolic dysregulation and chronic inflammation characteristic of the db/db brain rewire Rho GTPase signaling, potentially altering the role of *Arhgap15* in MG5‐associated phagocytic programs.

Furthermore, we observed a preferential transition of homeostatic microglia toward the DAM state in diabetic mice, particularly during early disease stages. This initial bias may support debris clearance [69], but persistent activation could contribute to later inflammatory and phagocytic remodeling. Regulatory‐network analyses nominated the *Spi1–Xbp1* transcriptional axis as a candidate regulator associated with this biased transition. Their co‐expression in primary microglia increased DAM‐associated markers, reduced IAM‐associated markers, and enhanced bioparticle uptake. These findings extend the established roles of *Spi1* in defining microglial identity and *Xbp1* in stress response [70, 71], supporting a role for their combined expression in DAM‐associated transcriptional remodeling.

Our cell‐cell communication analyses further nominated PROS1 *–* AXL as a candidate pathway associated with early DAM remodeling. Inferred PROS signaling in 12‐week‐old db/db mice, together with correlations between *Axl* expression and TF activity, suggests a possible link between intercellular signaling and the MG5 transcriptional state. Although *Axl* activation has been implicated in macrophage polarization toward a proinflammatory state through modulation of the MAPK and NF‐κB pathways [72], these observations do not establish a causal role for the proposed pathway in diabetic microglia. The technical limitations of the AAV pilot leave this relationship unresolved *in vivo*, warranting further study with a validated microglial delivery system or conditional genetic model.

In conclusion, this study maps changes in microglial states, transcriptional plasticity, and temporal dynamics in the db/db model of diabetes. Our findings support a working model linking early inflammatory and epigenomic priming to MG5‐associated phagocytic activation, biased HM‐to‐DAM transition, and accelerated DAM maturation. The proposed PROS1 *–*AXL pathway, *Spi1–Xbp1* axis, and *Arhgap15* represent candidate checkpoints for future mechanistic studies of diabetes‐associated neurodegenerative vulnerability.

Several limitations of our study should be acknowledged. Our analyses focused on purified microglia and did not directly assess neuronal survival or synaptic integrity. Accordingly, our conclusions are restricted to diabetes‐associated microglial states and regulatory programs linked to neurodegenerative vulnerability, rather than providing direct evidence of neuronal injury. The sequencing design included one mouse per genotype and time point, limiting animal‐level inference. In addition, the db/db model is defined by leptin receptor deficiency and an extreme metabolic phenotype that does not fully recapitulate the heterogeneity of human T2D, such that the microglial changes described here reflect the integrated effects of disrupted leptin signaling and systemic metabolic stress rather than hyperglycemia or insulin resistance alone. Future comparative studies using diet‐induced obesity and pharmacological T2D models, and direct validation in human diabetic brain tissue, once matched single‐cell datasets become available, will be important for extending and translating these findings.

## 4 Methods

### 4.1 Mice

Female db/db and db/m mice were obtained from the Laboratory Animal Services Centre at The Chinese University of Hong Kong (CUHK). The sequencing design included db/db and db/m mice at 12 and 60 weeks, together with intermediate db/db time points at 24, 36, and 48 weeks. Each genotype/time‐point sequencing condition was represented by one mouse. Independent validation cohorts were purchased from Guangdong Zhiyuan Biopharmaceutical Technology Co., Ltd. Flow‐cytometric validation included db/db and db/m mice at 12 weeks (*n* = 3 per genotype) and 24 weeks (*n* = 4 per genotype), whereas qRT‐PCR validation included db/db mice at 12 and 24 weeks (*n* = 4 per age). All animal procedures were approved by the Animal Experimentation Ethics Committee at CUHK [Ref No.: (19‐435) in DH/D/8/2/1 Pt.21].

### 4.2 Microglia Isolation and Purification

Mice were anesthetized intraperitoneally with a mixture of ketamine (75 mg/kg) and xylazine (10 mg/kg), followed by transcardial perfusion with ice‐cold phosphate‐buffered saline (PBS). The whole brain was dissected, and microglia were isolated according to a previously published protocol with minor modifications [73]. In brief, dissected brains were minced with a scalpel blade and enzymatically dissociated in DMEM/F12 medium containing dispase II, DNase I, and papain. The tissue was then mechanically dissociated by repeated pipetting through three differently sized glass pipettes. Cells that passed through a 40‐µm cell strainer were subjected to 30% Percoll gradient centrifugation. The resulting cell pellet was collected and washed twice with Hank's Balanced Salt Solution. Isolated microglia were further purified through fluorescence‐activated cell sorting (FACS) after Fc receptor blocking (BD Biosciences, BD553142) and labeling with conjugated antibodies CD11b‐FITC (BioLegend, 101205), CD45‐Pacific Blue (BioLegend, 103125), and TMEM119‐PE (Abcam, ab225496) on ice in the dark. Sytox Red (Invitrogen, S34859) was subsequently added, and CD11b^+^CD45^low^TMEM119^+^Sytox Red^−^ microglia were collected using a BD FACSMelody Cell Sorter (BD Biosciences).

### 4.3 Single‐Cell RNA Sequencing

Purified microglial cells were resuspended in PBS containing 0.04% bovine serum albumin before 10x Genomics 3' (v3.1) scRNA‐seq. Gene expression libraries were prepared following the manufacturer's protocol. In brief, cells were loaded onto a microfluidics chip using the 10x Controller to generate gel bead‐in‐emulsions (GEMs). Within each GEM, single cells and their transcripts were uniquely barcoded during reverse transcription of RNA into complementary DNA. Library construction included fragmentation, end repair, A‐tailing, double‐sided size selection, sample indexing, and PCR amplification. Quality control and assessment of the final libraries were performed using an Agilent Bioanalyzer DNA High Sensitivity chip, and sequencing was performed on an Illumina HiSeq X Ten at PE150.

### 4.4 Nucleus Isolation

The remaining cells from the same preparation (after scRNA‐seq) were used for nuclei isolation following the 10x Genomics low‐input protocol for snATAC‐seq, with minor modifications. In brief, the cell suspension was pelleted and lysed on ice in a buffer containing the detergents Tween‐20 and Nonidet P‐40. The isolated nuclei were then washed and resuspended in chilled 10× diluted nucleus buffer provided by 10x Genomics. Nucleus quality was microscopically examined after Trypan Blue staining before proceeding with downstream DNA library preparation and snATAC‐seq.

### 4.5 Single‐Nucleus ATAC Sequencing

Isolated microglial nuclei were processed using the 10x Genomics (v1.1) single‐cell ATAC‐sequencing platform. Chromatin accessibility libraries were prepared according to the manufacturer's protocol. In brief, nuclei suspensions were incubated in a transposition mix containing Tn5 transposase to generate adapter‐ligated DNA fragments corresponding to open chromatin regions. GEMs were then generated on a microfluidics chip, and single nuclei and DNA fragments were barcoded. Library construction included sample indexing and PCR amplification. Quality control and assessment of the final libraries were performed using an Agilent Bioanalyzer High Sensitivity DNA chip, and sequencing was performed on the Illumina NovaSeq 6000 at PE50.

### 4.6 Preprocessing of scRNA‐seq Data

Sequencing data were processed using CellRanger (v.6.0.0, chemistry V3). Raw reads were aligned to the GRCm39 (mm39) mouse reference genome to generate RNA count matrices, guided by GENCODE annotation [74] (M35). Quality control was performed to ensure data reliability for downstream analyses. Specifically, cells with fewer than 400 expressed genes, fewer than 500 detected UMIs, or over 20% mitochondrial gene content were considered low quality and removed from the dataset. Gene‐level filtering was permissive, retaining all genes detected in at least 20 cells. Potential doublets were identified and removed from each count matrix using DoubletFinder [75] (v3).

To obtain a comprehensive view of the cellular landscape, individual count matrices were imported into Seurat [76] (v 4.3.0) and merged into a single Seurat object for downstream analysis. The merged data were normalized using the SCTransform function, which applies a regularized negative binomial regression model to normalize gene expression and correct for technical variation by regressing out effects related to batch identity and mitochondrial gene percentage in each cell. Dimensionality reduction was performed using PCA via the RunPCA function with the top 2000 highly variable genes, followed by the *RunHarmony* function from the Harmony [77] package for additional batch correction. The top 30 principal components were then used to construct a uniform manifold approximation and projection (UMAP) using the *RunUMAP* function. A shared nearest neighbor graph was subsequently constructed using the *FindNeighbors* function (dims = 1:30) and used for Louvain community detection clustering via the *FindClusters* function (resolution = 0.2). Cluster‐specific marker genes were identified using the Wilcoxon rank‐sum test implemented in the *FindAllMarkers* function with the following parameters: only.pos = TRUE, logfc.threshold = 0.5, min.diff.pct = 0.3, and min.pct = 0.5.

### 4.7 Identification of DEGs

DEGs for each cell cluster between different genotypes and stages were identified using a multi‐method approach to ensure robustness and accuracy. Specifically, three methods were used: the Wilcoxon test built into Seurat, MAST [27], and limma‐trend [28]. Each method had its own set of cutoffs for defining DEGs. For the Wilcoxon test, genes with an absolute log_2_ fold change ≥ 0.36, expression in ≥ 40% of cells, and Benjamini–Hochberg adjusted *p*‐value < 0.01 were considered DEGs. For MAST, genes with an absolute coefficient ≥ 0.1 and false discovery rate (FDR) < 0.01 were considered DEGs. For limma‐trend, genes with an absolute log_2_ fold change ≥ 0.36 and FDR < 0.01 were considered DEGs. To increase confidence in the identified DEGs, only genes detected as DEGs by at least two of the three methods were retained for further analysis. Because MAST and limma‐trend do not report the proportion of cells expressing a candidate gene, DEGs expressed in fewer than 40% of cells, as reported by Seurat, were also removed.

### 4.8 AUCell‐Based Signature Scoring

Per‐cell enrichment of published and custom gene signatures was quantified using AUCell [78] (*AUCell_calcAUC*). Published signatures representing previously reported human and mouse microglial subtypes were obtained from the corresponding studies. The statistical significance of AUCell scores in each subpopulation was evaluated using the Wilcoxon rank‐sum test (*x* = AUC values within the subpopulation, *y* = AUC values in the remaining cells, alternative = greater). *p*‐values were adjusted for multiple hypothesis testing using the Benjamini–Hochberg procedure. In addition, a custom secreted neuroinflammatory and neuronal stress‐associated mediator signature was assembled from selected GO Biological Process categories and restricted to proteins annotated as secreted in the Human Protein Atlas. Human annotations were mapped to mouse orthologs where required. Statistical comparisons between the two genotypes were performed using the Wilcoxon rank‐sum test. The complete gene list and source annotations are provided in Table S14.

### 4.9 snATAC‐seq Data Processing and Analysis

Raw sequencing reads were aligned to the GRCm39 reference genome using the Cell Ranger ATAC pipeline (v2.1.0). Initial quality control was performed in ArchR [79] (v1.0.3) to retain nuclei (with TSS enrichment > 8, fragments per nucleus > 2500, and a blacklist ratio < 0.1). Doublets were identified and removed using the ArchR *addDoubletScores* and *filterDoublets* functions with default parameters to ensure high‐confidence single‐nucleus profiles. Dimensionality reduction was conducted using iterative latent semantic indexing through ArchR's *addIterativeLSI* module, followed by Seurat‐based Louvain clustering (resolution = 0.6) implemented in the *addClusters* function. Peak calling was performed using MACS2 [80] (v2.2.7) through ArchR's built‐in *addReproduciblePeakSet* function with default parameters to generate a consensus set of accessible regions. Cell embeddings and cluster annotations from ArchR and the reconstructed peaks‐by‐cell matrix were imported into Signac [81] (v1.14.0) for joint visualization and downstream analysis. DARs for each cell cluster between different genotypes and stages were identified using the Wilcoxon rank‐sum test implemented in Seurat, with significance thresholds set at absolute log_2_ fold change ≥ 0.36, accessibility in ≥ 40% of nuclei, and Benjamini–Hochberg adjusted *p*‐value < 0.01. Functional annotation of DARs was performed by linking peaks to putative target genes based on proximity and co‐accessibility, followed by pathway enrichment analysis to identify biologically relevant processes (Benjamini–Hochberg adjusted *p*‐value < 0.05).

### 4.10 Peak Annotation and Motif Enrichment Analysis

Peak annotation and motif enrichment were performed using HOMER [82] (v5.1). All peak coordinates were converted from the mm39 to the mm10 genome assembly using CrossMap [83] (v0.7.3) with the UCSC mm39ToMm10 chain file. Peak annotation was performed using HOMER's annotatePeaks.pl function, which assigns genomic features (e.g., promoter, exon, intron, and intergenic) and links peaks to the nearest genes. For motif enrichment analysis, selected peaks were analyzed using HOMER's findMotifsGenome.pl function using a 200‐bp window centered on each peak and the mm10 genome as the reference. Both known and de novo motif discovery were performed, with enrichment calculated relative to a random genomic background generated by HOMER to control for sequence composition bias.

### 4.11 Generation of Cis‐Co‐Accessibility Networks With Cicero

Cis‐co‐accessibility networks were predicted for all open regions in individual microglial clusters from snATAC‐seq libraries using Cicero [42] (v1.3.0). To ensure consistent penalization of long‐range interactions across all microglial clusters, we first aggregated all microglial cells and converted the combined peak‐by‐cell matrix into a Cicero CellDataSet object using the *make_cicero_cds* function, with UMAP coordinates from ArchR's dimensionality reduction as input to preserve cell‐state relationships. The global distance penalty parameter was then estimated using the *estimate_distance_parameter* function with default settings. The mean value of the estimated distance parameters was subsequently applied to each microglial cluster, which was separately converted into a Cicero CDS object for cell type–specific network inference. For each cluster, co‐accessibility scores between all pairs of accessible peaks within 500 kb were calculated using the run_cicero function to identify high‐confidence cis‐regulatory interactions.

To determine an optimal co‐accessibility threshold and control for background correlations in each microglial subset, we generated a null distribution by randomly permuting the columns of the cell‐by‐peak matrix and recalculating co‐accessibility scores. The empirical background was modeled as a normal distribution using the fitdist function from the fitdistrplus [84] R package, and significant co‐accessible pairs were defined as those with adjusted *p*‐values < 0.01 after Benjamini–Hochberg correction.

### 4.12 Trajectory Analysis

Developmental trajectories were inferred using Slingshot [85] (v2.6.0), a computational framework for reconstructing branching pseudotemporal trajectories from scRNA‐seq data. Cell clusters predefined in Seurat, along with their corresponding low‐dimensional UMAP embeddings, served as input for trajectory construction. The root cluster was manually specified based on previous biological knowledge, followed by the fitting of principal curves to delineate lineage relationships and branching points. Pseudotime values were then assigned to each cell along the inferred trajectories, representing their relative progression through the developmental process. Cells in the root cluster were assigned a pseudotime value of zero, and pseudotime increased monotonically along each lineage.

### 4.13 RNA Velocity Analysis

RNA velocity analysis was performed using Velocyto [86] to gain insights into the dynamic states of cells. Velocyto quantifies unspliced (nascent) and spliced (mature) mRNA molecules, with the ratio between the two serving as an estimate of the future state of the gene expression profile. Velocyto was run for each sample using the run10x command on CellRanger outputs to generate loom files containing spliced and unspliced mRNA counts. The resulting loom files were then loaded into Python and processed with scVelo [87] for quality control, normalization, and RNA velocity estimation.

### 4.14 Pseudotime Gene Expression Clustering

Pseudotime‐based gene expression clustering was performed to resolve lineage‐specific transcriptional dynamics. Genes were first subjected to expression‐based quality filtering, and those with normalized expression > 0.5 in at least 25% of cells within any cluster or lineage were retained. To minimize technical noise, cells within each lineage were stratified into 20 pseudotime bins, and bin‐wise mean expression values were computed for each gene. Subsequently, pseudotime‐resolved Pearson correlation coefficients (PPCCs) and root mean square error (RMSE) values were calculated for each gene between Lineage 2 and either Lineage 1 or 3 based on binned expression profiles. Genes showing weak correlation with both comparator lineages (absolute PPCC < 0.4) and large deviation (RMSE above the 75th percentile in both comparisons) were retained and partitioned into distinct clusters according to their pseudotemporal expression patterns using the pam function implemented in the R package cluster.

### 4.15 Single‐Cell hdWGCNA Analysis

The hdWGCNA [54] package was used to perform gene co‐expression network analysis of MG5 cells. The Seurat object was prepared for hdWGCNA by subsetting the data to include genes expressed in at least 5% of cells. Metacells were constructed using the MetacellsByGroups function with the following parameters: k = 25, assay = SCT, max_shared = 10, min_cells = 50, reduction = harmony, and slot = counts. A topological overlap matrix was then computed, followed by hierarchical clustering to identify co‐expression modules using the onstructNetwork function with a soft‐power threshold of 14. Modules were defined using the dynamic tree cut algorithm with a minimum module size of 50 genes. Module eigengenes, representing the first principal component of each module, were calculated using the ModuleEigengenes function to summarize module expression profiles.

### 4.16 kNN‐DREMI Analysis

To quantify the strength and directionality of gene regulatory relationships, we applied kNN‐DREMI [88], a nonparametric method for estimating mutual information between gene pairs conditioned on cellular states. Specifically, the SCTransform‐normalized count matrix for candidate cells was preprocessed by normalizing and scaling gene expression values using the *scprep.normalize.library_size_normalize function* (scprep v1.2.3). To address the sparsity and noise inherent in scRNA‐seq data, data imputation and denoising were performed using MAGIC (v3.0.0). The *fit_transform* function from MAGIC was applied with parameters set to n_pca = 100, k = 20, and t = 5 to preserve global data structure while enhancing local gene–gene relationships. For kNN‐DREMI analysis, we used the *knnDREMI* function implemented in MAGIC, with parameters k = 5, n_bins = 50, and n_mesh = 5 for kernel density estimation.

### 4.17 Functional Enrichment and Pseudotime‐Resolved GSEA

Functional enrichment analysis was conducted for each gene set using the *enrichKEGG* and *enrichGO* functions from the clusterProfiler [89] package (v4.8.3) in R. KEGG pathways and GO terms with Benjamini–Hochberg adjusted *p*‐values below 0.05 were considered significantly enriched. To identify gene sets exhibiting coordinated expression dynamics along pseudotime trajectories, GSEA was performed in a pseudotime‐resolved manner. Specifically, a Pearson correlation was calculated between gene expression and cell pseudotime for each gene in each cell cluster or trajectory lineage. Genes were then ranked by their corresponding Pearson correlation coefficients, which served as a continuous phenotype for GSEA, implemented through the GSEA function in clusterProfiler. Gene sets with a Benjamini–Hochberg adjusted *p*‐value < 0.1 were considered significant. Disease‐associated gene set enrichment was evaluated using the gseDGN function from the DOSE [90] package (v3.26.2), leveraging curated disease–gene associations from the DisGeNET database.

### 4.18 Flow‐Cytometric Analysis of Microglial States

Live singlet CD11b^+^CD45^low^TMEM119^+^ cells from independent cohorts of 12‐ and 24‐week‐old db/db and db/m mice were gated as microglia. Microglial states were assessed using fluorophore‐conjugated antibodies against CLEC7A (Invitrogen, 12‐5859‐80), P2RY12 (BioLegend, 848005), and CD83 (BioLegend, 121517). For quantification of the MG5‐associated population, a common CLEC7A^high^ threshold was defined from the extreme upper tail of the pooled 12‐week‐old db/m reference distribution and applied unchanged to all samples. CLEC7A^high^ cells were quantified as a percentage of the parent microglial population.

For analysis of HM transitional populations, a P2RY12^high^, HM‐lineage‐enriched compartment was first selected. Fixed CLEC7A‐vs.‐CD83 quadrant gates then classified P2RY12^high^CLEC7A^high^CD83^low^ cells as HM‐DAM‐like, P2RY12^high^CLEC7A^low^ CD83^high^ cells as HM‐IAM‐like, and double‐low cells as remaining HM. Dual‐high cells were retained in the parent denominator but were not assigned to either branch. Gates were applied unchanged across samples, and frequencies were calculated relative to all P2RY12^high^ microglia using FlowJo (v10). Genotype differences were evaluated using a two‐sided Welch's *t*‐test.

### 4.19 Phagocytosis Assay in Primary Microglia

Primary mouse microglia were purchased from Shanghai Jinyuan Biotechnology Co., Ltd. (JY‐J659) and maintained in DMEM/F12 (Gibco, 11320) at 37°C in a humidified atmosphere containing 5% CO_2_ according to the manufacturer's instructions. The *Arhgap15* expression construct, dual *Spi1‐Xbp1* expression construct, *Arhgap15*‐targeting shRNA, empty vector control, and non‐targeting shRNA controls were purchased from Beijing AIVD Biotechnology Co., Ltd. Cells were transduced with the respective lentiviral vectors, cultured for 4 days, and subsequently incubated for 1 h at 37°C with pHrodo Green *E*. *coli* BioParticles (Invitrogen, P35366). After incubation, cells were washed with PBS and immediately processed for flow cytometry. Phagocytic activity was quantified as the percentage of fluorescein isothiocyanate‐positive cells using a BD FACSVerse flow cytometer (BD Biosciences). The acquired data were analyzed using FlowJo software (v10).

### 4.20 RNA Extraction and qRT‐PCR

Total RNA was isolated from freshly isolated brain microglia or cultured primary microglia using RNAiso Plus (Takara, 9108) and RNeasy Mini kit (Qiagen, 74106), followed by cDNA synthesis using the PrimeScript RT Master Mix Kit (Takara, RR036A). Each qRT‐PCR reaction was performed in triplicate using TB Green Premix Ex Taq II (Takara, RR820A). Gene expression was normalized to the endogenous control *Gapdh*. All primer sequences are listed in Table S15.

### 4.21 Cell Fate Commitment Analysis

CytoSimplex [57] was used to project single cells onto a ternary simplex, where the vertices represent predefined terminal states. Cell coordinates derived from the expression profiles of subset‐specific DEGs quantitatively represent the transcriptional affinity toward each fate.

### 4.22 TF Regulatory Network Inference

TF regulatory networks were inferred from SCTransform‐normalized gene expression data using pySCENIC [58] (v0.11.2). Initial data filtering was performed to remove sparsely expressed genes detected in fewer than 1% of cells. Co‐expression modules (regulons) were then inferred using the grn function, which generated an adjacency matrix from the gene count matrix supplemented with a comprehensive list of mouse‐specific TFs (available at https://resources.aertslab.org/cistarget/tf_lists/allTFs_mm.txt). These modules were subsequently refined using the ctx function, which excluded target genes lacking enrichment for TF‐specific motifs, leveraging a mouse‐specific motif database (https://resources.aertslab.org/cistarget/motif2tf/motifs‐v10nr_clust‐nr.mgi‐m0.001‐o0.0.tbl) and cis‐regulatory annotations (mm10_500bp_up_100bp_down_full_tx_v10_clust.genes_vs_motifs.rankings.feather, mm10_10kbp_up_10kbp_down_full_tx_v10_clust.genes_vs_motifs.rankings.feather, available from https://resources.aertslab.org/cistarget/databases/mus_musculus/mm10/refseq_r80/mc_v10_clust/gene_based/). Finally, aucell was used to calculate per‐cell enrichment scores for each regulon, quantifying TF activity across individual cells.

### 4.23 Gene Perturbation Simulation

Gene activation and knockout simulations were performed using dynamo [59] (version 1.4.1). The analysis began with the construction of an AnnData object using the SCTransform‐normalized gene expression matrix, together with spliced and unspliced expression matrices generated by Velocyto. Dimensionality reduction embeddings, including PCA and UMAP computed by Seurat, were integrated into the AnnData object to facilitate trajectory inference. The *dyn.pp.Preprocessor* function was applied to filter cells and genes, retaining the top 3,000 highly variable genes using the built‐in Monocle3 recipe. For gene activation simulations, the target gene's expression was computationally elevated to a value of 100 across all cells, whereas for knockout simulations, the gene's expression was set to −100. These perturbations were designed to mimic strong overexpression and complete loss‐of‐function scenarios, respectively. The *dyn.pl.perturbation* function was then used to recalculate RNA velocity vectors, enabling the assessment of changes in transcriptional dynamics following each perturbation. The resulting velocity shifts were visualized in UMAP space to evaluate the effect of perturbations on cellular trajectories. The RNA Jacobian matrix was computed using the *dyn.vf.Jacobian* function with default parameters to quantify regulatory interactions between genes.

### 4.24 Cell–Cell Communication Analysis

Cell–cell communication networks were inferred using CellChat [63] (v1.6.0). CellChat uses a curated database of ligand–receptor pairs (signaling pathways) to infer communication probabilities between cell types, accounting for both ligand and receptor expression levels as well as their interaction specificity. Outgoing and incoming signaling pathways were identified and quantified based on differentially overexpressed ligands and receptors within each cell group, following the official documentation with default parameters. To compare intercellular communication across conditions, individual CellChat objects from each dataset were merged to visualize differences in communication strength. Differential outgoing and incoming signaling pathways between db/db and db/m were identified using the *netAnalysis_signalingChanges_scatter* function, providing insights into condition‐specific alterations in cell–cell communication networks.

### 4.25 AAV Design, Quality Control, and In Vivo Administration

A single‐stranded AAV transfer vector was adapted from previously published frameworks reported to enrich transgene expression in microglia [66, 67]. The published reporter configuration was modified by incorporating EGFP and the *Axl*‐targeting sequence TRCN0000023312 (CCAGTCAAGTGGATTGCTATT) in a miR‐30‐based cassette [91]; the matched control vector contained a non‐targeting sequence. AAV‐shNC and AAV‐shAxl were produced by Beijing AIVD Biotechnology Co., Ltd., with qPCR‐determined titers of 1.45 × 10^1^ ^3^ and 1.20 × 10^1^ ^3^ vg/mL, respectively, and showed no prominent contaminating protein bands by SDS‐PAGE. Ten‐week‐old db/m and db/db mice received bilateral lateral‐ventricle injections of AAV‐shNC or AAV‐shAxl (2.5 µL and approximately 3 × 10^10^ vg per ventricle; anteroposterior −0.5 mm, mediolateral ±1.1 mm, dorsoventral −3.0 mm). The behavioral cohort comprised db/m‐shNC (*n* = 3), db/m‐shAxl (*n* = 3), db/db‐shNC (*n* = 5), and db/db‐shAxl (*n* = 5) mice. Behavioral testing and terminal tissue collection were performed at 20 weeks.

### 4.26 Behavioral Assessment

At 20 weeks of age, open‐field activity was recorded for 600 s, and total distance traveled, mobile time, and center entries were quantified. Y‐maze behavior was recorded for 300 s; total arm entries were used as a locomotor/exploration measure, and spontaneous alternation was calculated only for mice with at least three total arm entries. Novel‐object‐recognition (NOR) sessions were recorded for 300 s. Total object‐investigation time was analyzed as an exploratory engagement measure. Data are shown as mean ± SD, with each point representing one mouse. AAV‐shNC and AAV‐shAxl groups were compared within each genotype using two‐sided unpaired Welch's *t*‐tests.

### 4.27 Immunofluorescence Assessment

Following behavioral testing at 20 weeks, mice were transcardially perfused with phosphate‐buffered saline, and brains were fixed in 4% paraformaldehyde and cryosectioned coronally at 30 µm. Free‐floating sections were immunostained for GFP and Iba1 and mounted with DAPI‐containing medium. Images were acquired using a 20× objective on an EVIDENT fluorescence microscope from regions spanning the lateral ventricle to hippocampal CA2. GFP, Iba1, and DAPI channels were combined to generate the merged images.

### 4.28 Statistical Analysis

Statistical analyses were performed using R (v4.3.1). Experimental data are presented as mean ± SD, with individual data points representing independent mice or independent cell‐culture experiments. For scRNA‐seq and snATAC‐seq analyses, cell‐level Wilcoxon rank‐sum tests were used for exploratory comparisons. Benjamini–Hochberg correction was applied where multiple genes, pathways, or signatures were tested. For qRT‐PCR, relative expression was calculated using the 2^−ΔΔCt^ method, while statistical testing was performed on ΔCt values. Independent mouse cohorts were compared using two‐sided unpaired Welch's *t*‐tests, whereas matched control and treatment conditions from the same primary‐microglia experiment were compared using two‐sided paired *t*‐tests. Flow‐cytometric comparisons between db/db and db/m mice were performed using two‐sided unpaired Welch's *t*‐tests. Pearson and Spearman correlation tests were two‐sided. Statistical significance was determined using analysis‐specific thresholds, as specified in the corresponding Methods subsections and figure legends. The statistical test, data presentation, sample size, and significance annotations are provided in the corresponding figure legends.

## Author Contributions

J.Z., J.T., and T.F.C. conceived and designed the study. J.T. generated the sequencing data. J.Z. performed the primary computational data analysis, with contributions from T.K.Y. Y.J.Z., J.Z., and T.K.Y. designed the experiments. T.K.Y. and Y.J.Z. performed the primary experimental validation, assisted by T.H.L. and D.H. T.F.C. supervised the entire project. J.Z., T.K.Y., and J.T. wrote the original manuscript draft. All authors reviewed and edited the final manuscript.

## Ethics Statement

All animal procedures described herein were approved by the Animal Experimentation Ethics Committee at CUHK.

## Conflicts of Interest

The authors declare no conflicts of interest.

## Supporting information




**Supporting File 1**: advs78509‐sup‐0001‐SuppMat.docx.

**Supporting File 2**: advs78509‐sup‐0002‐tables S1‐S15.xlsx.

## Data Availability

The raw sequence data generated in this paper have been deposited in the Sequence Read Archive under the accession number PRJNA1358530.
