# Supplementary material for: Single‐Cell Dissection of Microglial Dysfunction in Diabetes‐Associated Neurodegenerative Vulnerability
Source: Adv Sci (Weinh). 2026 Oct 28:e78509. Online ahead of print. doi: 10.1002/advs.202600023 (PMC13715674; doi:10.1002/advs.202600023)
Supplement: Supplementary file 1 [file ADVS-9999-e78509-s001.docx]

**Figure S1.** **Quality control and preprocessing of single-cell RNA-seq data.**

**(A)** Representative flow cytometry plots showing the isolation strategy for mouse microglia. Cells were sequentially gated for singlets, CD45^low^ cells, CD11b^high^ cells, TMEM119^+^, and Sytox Red⁻. Numbers indicate the percentage of cells in each gate. **(B)** UMAP embeddings of all single cells profiled using scRNA-seq, colored by mitochondrial gene ratio, gene count, and UMI count. **(C)** UMAP embedding of all profiled cells with computationally predicted doublets highlighted in red, identified using DoubletFinder. **(D)** UMAP embedding of all profiled cells with low-quality cells (high mitochondrial content, low gene or UMI count, or predicted doublet status) highlighted in red; these cells were excluded from downstream analyses. **(E)** Scatter plot illustrating the correlation between UMI and gene counts after quality control. Each point represents a cell, colored by the mitochondrial gene ratio; the Pearson correlation coefficient (r) and two-sided P value are indicated. **(F)** UMAP embeddings before (left) and after (right) batch correction using Seurat and Harmony, colored by sample origin.

**Figure S2. Clustering and annotation of single-cell transcriptomic profiles from the mouse brain.**

**(A)** UMAP embedding of all cells captured using scRNA-seq after quality control, colored by cell clusters identified using the Seurat pipeline. **(B)** Bar plots showing the composition of cell clusters in each dataset. **(C)** Dot plot illustrating the scaled average expression (color) and percentage of expressing cells (dot size) for canonical microglial markers across microglial clusters (1–11) and representative marker genes for nonmicroglial cell types. **(D)** Ridge plot illustrating the distribution of the UMI-to-gene count ratios in each Seurat-defined cell cluster. **(E)** Representative enriched Gene Ontology biological processes for each microglial transcriptional state. **(F)** Heatmap showing the scaled average expression of representative TFs in each microglial transcriptional state. **(G)** UMAP embeddings colored by the DAM signature score from Keren-Shaul et al. and the MGnD signature score from Krasemann et al. **(H–J)** Cross-study comparison of MG1–MG11 with microglial states reported by Dong et al. (H), Yaqubi et al. (I), and Sun et al. (J), based on representative signature gene sets for each cluster. Each point represents the mean scaled signature score for a given published cluster’s gene set in each microglial state from this study. Dots indicate statistically significant enrichment (Wilcoxon rank-sum test with Benjamini–Hochberg correction; adjusted P < 0.01).

**Figure S3. Comparative analysis of aging-associated transcriptional and metabolic changes in microglial states.**

**(A and B)** Venn diagrams showing the number of aging-associated DEGs in each microglial state for (**A**) db/db and (**B**) db/m mice. DEGs were identified using the Wilcoxon test, MAST, and limma-trend, with overlaps indicating shared or state-specific changes. **(C and D)** Pairwise comparison of aging-associated DEGs across microglial states for (**C**) db/db and (**D**) db/m mice. Bar plots show the Jaccard index (overlap magnitude), and statistical significance was examined using Fisher’s exact test (Benjamini–Hochberg adjusted; *** adjusted P < 0.001). **(E)** PCA of pseudobulk gene expression per Seurat cluster, illustrating sample variance by genotype (db/db vs. db/m) and time point (12 weeks vs. 60 weeks). **(F)** Cumulative distribution of Δlog_2_FC values (log_2_FC[db/db 60 weeks vs. 12 weeks] - log_2_FC[db/m 60 weeks vs. 12 weeks]; x-axis) for genes in microglial states corresponding to gene clusters 1 and 2 (as shown in Figure 1G). The *y* axis shows the cumulative proportion of genes; each line represents a distinct microglial transcriptional state. **(G)** PCA of pseudobulk metabolic flux intensity, highlighting variance by genotype and time point. **(H)** Density plot showing the distribution of the difference in average scaled metabolic flux between 60 and 12 weeks for db/db and db/m mice. **(I)** Cell-level AUCell scores for a curated signature of secreted neuroinflammatory and neuronal stress-associated mediators in MG1–MG8, compared between db/db and db/m cells. Boxes show medians and interquartile ranges; genotype comparisons used Wilcoxon rank-sum tests with Benjamini–Hochberg correction. **adjusted P < 0.01 and ***adjusted P < 0.001. N.S., not significant.

**Figure S4. Genotype-associated transcriptional remodeling in microglial states during aging.**

**(A and B)** Venn diagrams showing the number of genotype-associated DEGs in each microglial state for (**A**) 12-week and (**B**) 60-week-old mice. DEGs were identified using the Wilcoxon test, MAST, and limma-trend, with overlaps indicating shared or state-specific transcriptional changes. **(C and D)** Pairwise comparison of genotype-associated DEGs across microglial states for (**C**) 12-week and (**D**) 60-week-old mice. Bar plots show the Jaccard index (overlap magnitude), and statistical significance was examined using Fisher’s exact test (Benjamini–Hochberg adjusted; *** indicates adjusted P < 0.001). **(E)** Scatter plot comparing the proportion of DEGs per microglial state (relative to total DEGs) at 12 weeks (*x* axis) vs. 60 weeks (*y* axis). The diagonal line indicates equal proportions. **(F)** Bar plot showing the composition of microglial clusters in db/db and db/m mice at 12 and 60 weeks, highlighting genotype- and age-dependent shifts. **(G)** Scatter plot showing the concordance of genotype-associated DEG expression changes in MG5–MG8 between db/db and db/m mice at 12 and 60 weeks. Each point represents a gene, colored by shared or time point–specific DEGs; the Pearson correlation coefficient (r) and two-sided P-value indicate the degree of consistency. **(H)** Heatmap showing Jaccard indices comparing genotype-associated DEGs (per cluster at 12 and 60 weeks) with aging-associated DEGs in db/m mice (60 weeks vs. 12 weeks). No comparisons were significant (Fisher’s exact test with Benjamini–Hochberg correction). **(I)** Pearson correlation between log_2_FC values for genotype-associated DEGs (*x* axis) and aging-associated DEGs in db/m mice (*y* axis), evaluating the directionality of expression changes.

**Figure S5. Quality control and preprocessing of single-nucleus ATAC-seq data.**

**(A-D)** Violin plots displaying quality metrics for snATAC-seq data in db/db and db/m mice at 12 and 60 weeks: (**A**) TSS enrichment scores, (**B**) number of unique fragments per nucleus, (**C**) blacklist region ratio, and (**D**) doublet enrichment scores. **(E)** Frequency distribution plots showing fragment size distributions for each snATAC-seq dataset. **(F)** Density scatterplot illustrating fragments per nucleus and individual TSS enrichment scores. Nuclei in the top right quadrant (TSS enrichment > 8 and >2,500 fragments per nucleus) were retained for subsequent quality control. **(G)** Bar plot showing the number of nuclei retained after each quality control step, including filtering for TSS enrichment, fragment count, blacklist ratio, and doublet exclusion. **(H)** Bar plot showing the number of nuclei retained after quality control for each snATAC-seq dataset. **(I)** UMAP embeddings of all nuclei passing quality control before (left) and after (right) batch correction using ArchR and Harmony, colored by sample origin.

**Figure S6. Characterization of microglial epigenetic states defined by snATAC-seq data.**

**(A)** UMAP embedding of all nuclei captured by snATAC-seq after quality control, colored by cell clusters identified using the ArchR pipeline. **(B)** Dot plot showing the scaled average gene activity (color) and fraction of nuclei with detected activity (dot size) for canonical microglial markers across microglial clusters (ATAC-1 to ATAC-10) and representative markers of nonmicroglial cell types. **(C)** Heatmap of Jaccard indices comparing marker genes between snATAC-seq and scRNA-seq microglial populations. Statistical significance was examined using Fisher’s exact test (Benjamini–Hochberg adjusted; * indicates adjusted P < 0.05). **(D)** UMAP embeddings showing the joint integration of RNA and ATAC data using ArchR, with unconstrained (left) and constrained (right) integration, colored by Seurat-defined RNA clusters. **(E)** Genomic annotation of accessible chromatin peaks in microglial nuclei, showing the fraction of peaks overlapping with annotated ENCODE cCREs. **(F)** Overlap of accessible peaks with previously reported cCREs detected in the mouse brain and cerebrum. **(G)** Bar plot showing the distribution of DARs across all ATAC microglia clusters. Left: DARs distal to promoter-TSS regions (HOMER annotation); Right: DARs overlapping promoter-TSS regions. **(H)** Bar plot showing the number of co-accessible cCRE pairs identified in each ATAC microglial cluster, categorized by proximal–proximal, proximal–distal, and distal–distal interaction. Proximal peaks are defined as those annotated as promoter-TSS regions by HOMER; distal peaks represent all other cCREs. **(I)** Density plot showing summit distances between peaks in proximal–distal co-accessible pairs, with a mean distance of 144 kb. **(J)** Distribution of the number of co-accessible cCRE pairs detected across all ATAC microglial clusters. **(K)** Representative KEGG pathways enriched among genes whose promoter-TSS regions exhibit significant co-accessibility with II-DARs.

**Figure S7. Recurrence and reversibility of microglial gene expression changes across the aging trajectory in db/db mice.**

**(A)** Bar plots showing the absolute number of upregulated (top) and downregulated DEGs (bottom) identified between adjacent time points for each scRNA-seq-defined microglial state in db/db mice. Only clusters with more than 10 cells per condition were included in the DEG analysis. **(B)** Distribution of DEG recurrences across all microglial clusters for sequential comparisons: 36 weeks vs. 24 weeks, 48 weeks vs. 36 weeks, and 60 weeks vs. 48 weeks. **(C)** Distribution of DEG recurrences across sequential comparisons within MG2–MG8 microglial clusters. **(D)** Pearson correlation coefficients quantifying the concordance of temporal transcriptional dynamics calculated separately for each microglial cluster. For each cluster, the log_2_FC values for all DEGs identified in either comparison were used to determine the correlation. *P < 0.05, **P < 0.01, ***P < 0.001. **(E)** Heatmap of Jaccard indices comparing upregulated genes in “36 weeks vs. 24 weeks” with downregulated genes in “48 weeks vs. 36 weeks” across microglial states. Statistical significance was examined using Fisher’s exact test (Benjamini–Hochberg adjusted; * indicates adjusted P < 0.05). **(F)** Representative enriched GO terms for overlapping genes between upregulated genes in “36 weeks vs. 24 weeks” and downregulated genes in “48 weeks vs. 36 weeks”. **(G)** Dot plot showing the average gene signature scores for human phenotype–associated gene sets, as defined by the Human Phenotype Ontology, across individual db/db samples at each time point.

**Figure S8. Trajectory validation and co-expression network analysis reveal coordinated gene programs in DAM.**

**(A)** UMAP embedding showing RNA velocity analysis performed using scVelo with velocity vectors overlaid on individual cells. **(B)** Heatmap showing pseudotemporal gene expression patterns along the three lineages identified using Slingshot. Genes (rows) were hierarchically clustered based on smoothed expression profiles across pseudotime bins (columns) for each lineage. Color intensity indicates z-scored expression, and the line plot displays smoothed expression activity along the trajectory. **(C)** Hierarchical clustering dendrogram of co-expressed genes in DAM cells, with color bars indicating module assignments from hdWGCNA analysis. **(D)** Dot plot showing module eigengene (ME) score across 11 microglial states. Color indicates the scaled average ME score, and dot size indicates the fraction of cells expressing module genes. **(E)** hdWGCNA-derived co-expression networks for modules M5 (top) and M9 (bottom) in DAM cells, showing the top 25 genes ranked by eigengene connectivity.  **(F)** Representative enriched KEGG pathways for genes belonging to modules M5, M6, and M9. **(G)** Heatmap showing the overlap between trajectory-induced gene clusters (G1–G8; columns) and hdWGCNA gene modules (M1–M14; rows). Color intensity represents the number of overlapping genes, and dot size indicates the proportion of overlapping genes relative to the size of trajectory-induced clusters. Significant overlaps (Fisher’s exact test, Benjamini–Hochberg adjusted P < 0.05) indicate convergence between temporal and co-expression-based gene groupings. **(H)** Single-cell kNN-DREMI analysis illustrating the relationship between the expression of Arhgap15 and selected phagocytosis-associated genes after MAGIC imputation. **(I)** qRT-PCR validation of *Arhgap15* overexpression, knockdown, and rescue in primary mouse microglia. Expression was normalized to Gapdh and calculated relative to the mean of the corresponding control group. Bars show mean ± SD, and points represent independent paired cell-culture experiments (n = 3). Statistical comparisons were performed on ΔCt values using two-sided paired t-tests. *Arhgap15* transcript abundance modestly exceeded that in the rescue control, indicating a slight over-restoration of expression. Despite this modest increase, no significant differences were detected between the rescue and matched-control groups in pHrodo uptake or the downstream phagocytosis-associated markers examined. *P < 0.05; **P < 0.01; ***P < 0.001.

**Figure S9. Bifurcated activation trajectories and regulatory programs of homeostatic microglia in db/db mice.**

**(A)** Ternary plot showing the distribution of HM, DAM, and IAM populations in each sample based on subtype-specific gene signatures, overlaid with RNA velocity vectors to visualize transcriptional state transitions. **(B)** Expression of *Clec7a*, *Cd83*, and *P2ry12* in HM-DAM, HM-IAM, and remaining HM cells. Dot color indicates mean expression, and dot size indicates the percentage of cells expressing each gene. (C) Left, representative CLEC7A-versus-CD83 flow cytometry plots of P2RY12^high^ microglia from 24-week-old db/m and db/db mice. Fixed quadrant gates classify P2RY12^high^CLEC7A^high^CD83^low^ and P2RY12^high^CLEC7A^low^CD83^high^ cells as HM-DAM-like and HM-IAM-like, respectively. Right, frequency of HM-IAM-like cells among all P2RY12^high^ microglia at 12 and 24 weeks. Bars show mean ± SD, and points represent independent mice (12 weeks, n = 3 per genotype; 24 weeks, n = 4 per genotype). Statistical testing used a two-sided unpaired Welch’s t-test; ***P < 0.001. **(D)** Volcano plot showing differential gene expression between HM-DAM and HM-IAM states. DEGs (Seurat Wilcoxon test; |log_2_FC| ≥ 0.36, adjusted P < 0.01) are colored by direction of dysregulation (red: upregulated in HM-DAM and blue: downregulated in HM-DAM/upregulated in HM-IAM). Representative candidate genes are annotated. **(E and F)** Representative enriched KEGG pathways for (E) upregulated and (F) downregulated genes in HM-DAM cells. Upregulated genes showed dominant enrichment in neurodegenerative disease pathways, whereas downregulated genes were enriched in signaling pathways mediating acute inflammatory responses. **(G)** Scatter plot showing the proportion of HM-DAM cells across 50 evenly sized pseudotime bins. The Pearson correlation coefficient (r) and two-sided P-value are indicated. **(H)** Volcano plot showing differential TF activity between HM-DAM and HM-IAM states. TFs with biased activity (|log_2_FC| ≥ 0.2, adjusted P < 0.01) are colored by direction of activation (red: active in HM-DAM and blue: active in HM-IAM). TFs showing increased activity in HM-DAM cells are labeled. **(I)** Ranking of TFs in HM-DAM (left) and HM-IAM (right) cells based on their regulon specificity scores derived from SCENIC analysis, indicating the degree to which each TF’s activity is specific to either the HM-DAM or HM-IAM trajectory. **(J)** kNN-DREVI plots relating *Spi1* or *Xbp1* expression to *Cd63*, *Clec7a*, and *Ctsb* expression after MAGIC imputation; DREMI values are shown. **(K)** kNN-DREVI plot relating *Spi1* and *Xbp1* expression after MAGIC imputation. **(L)** Overlap analysis of *Spi1* and *Xbp1* target genes. Statistical significance was examined using Fisher’s exact test. **(M)** qRT-PCR validation of *Spi1* and *Xbp1* co-expression in primary mouse microglia. Expression was normalized to Gapdh and calculated relative to the mean of the corresponding control group. Bars show mean ± SD, and points represent independent paired cell-culture experiments (n = 3). Statistical comparisons were performed on ΔCt values using two-sided paired t-tests. ***P < 0.001.

**Figure S10. Technical evaluation of an AAV approach intended for microglial *Axl* knockdown.**

**(A)** AAV design adapted from a published framework for microglia-enriched transgene expression, incorporating EGFP and a miR-30-based shAxl cassette. **(B)** Open-field, Y-maze, and novel-object-recognition measurements in db/m and db/db mice receiving AAV-shNC or AAV-shAxl. Bars show mean ± SD, and points represent individual mice (db/m, n = 3 per treatment group; db/db, n = 5 per treatment group). Y-maze spontaneous alternation included only mice with at least three total arm entries. AAV-shNC and AAV-shAxl groups were compared within each genotype using two-sided unpaired Welch’s t-tests; N.S., not significant. **(C)** GFP, Iba1, DAPI, and merged fluorescence images from four individually identified 20-week-old db/db mice receiving AAV-shNC or AAV-shAxl, showing regions spanning the lateral ventricle to hippocampal CA2. A magnified view of the indicated region is also shown. Detectable GFP-positive profiles were predominantly observed in Iba1-negative cells in both vector groups. Scale bars, 100 μm. This technical pilot did not meet the requirements for in vivo functional validation: the vector did not achieve effective microglial targeting, and *Axl* knockdown was not verified. The behavioral findings therefore cannot be interpreted as evidence for or against a causal role for *Axl* in vivo.
